# β-suppressor protein 1 (ARRB1)-△exon13 modulates the progression of glioblastoma via combination with glycolysis-related proteins

**DOI:** 10.1016/j.bbrep.2025.102048

**Published:** 2025-05-13

**Authors:** Zi-Long Wei, Shuo Han, Dong-Hua Han, Xue-Tao Li, Yu-Lun Huang, Zhi-Min Wang

**Affiliations:** a Department of Neurosurgery, The Fourth Hospital Affiliated to Soochow University, Suzhou Dushu Lake Hospital, Suzhou, 215000, China; b Department of Neurosurgery, Shanghai Pudong Hospital Affliated to Fudan University, Pudong Medical Center, Shanghai, 201399, China; c Department of Neurosurgery, Shanghai Changzheng Hospital, Shanghai, China

**Keywords:** Glioblastoma, ARRB1, Alternative spicing, Glycolysis, Malignancy

## Abstract

Glioblastoma multiform (GBM) constitutes approximately 14.7 % of all central nervous system tumors (CNSTs) and 45.2 % of primary malignant CNSTs. Extensive research has indicated that β-arrestin 1 (ARRB1) plays a significant role in tumor malignancy. In this investigation, we established GBM cell lines representing normal control (NC), overexpression (OE) and Δexon13 GBM variants (△exon13) of ARRB1. Our findings indicate that the ARRB1-OE isoform facilitated GBM cell proliferation and migration, with the ARRB1-△exon13 isoform further augmenting this effect. Notably, the isoform ARRB1-△exon13 binds to glycolytic proteins including ENO1 and ALDOA and regulates glycolysis. In vivo studies corroborate the tumor-promoting effects of ARRB1-Δexon13. Furthermore, we demonstrate that 2-DG effectively inhibits the malignancy-promoting capabilities of ARRB1-Δexon13 by reducing pyruvate levels. Our identification of alternative RNA splicing events of ARRB1 reveals a mechanism by which GBM cell malignancy is augmented through ARRB1-Δexon13, which mediates glycolysis-related pathways.

## Highlights

• The first study to explore mechanism of ARRB1-△exon13 regulating tumor growth and migration via mediating glycolysis.
• ARRB1-△exon13 serves to promote the proliferation and invasion of glioblastoma cells.
• Targeting ARRB1-△exon13 is a new anti-tumor strategy in glioma which is reliant upon glycolysis as an energy source.

## Graphical abstract

**Figure:**
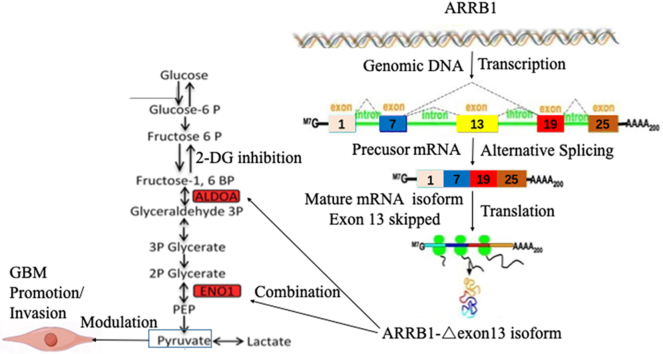

## 1 Introduction

Glioblastoma multiform (GBM) is the most prevalent and aggressive primary brain tumor, characterized by a poor median survival of 15 months [1]. It has been observed that GBM undergoes aerobic glycolysis, leading to the production of anabolic metabolites [2], and these metabolites could be utilized by neighboring GBM cells to facilitate ATP production via oxidative phosphorylation [3]. Yet, the precise molecular mechanisms by which aerobic glycolysis influences GBM progression require more investigation [4].

Increasing reports suggest that alternative splicing (AS) events represent a largely unexplored source of neoantigens for tumors [5]. The variability of tumor antigens due to tumor-specific AS affect immunotherapy outcomes and patient prognosis [6]. While AS has been documented in various tumors [7], the regulatory mechanisms of AS in β-arrestin1 (ARRB1) that contribute to malignant phenotypes in GBM, including invasiveness, growth and aberrant metabolism remain poorly understood [8].

ARRB1 was initially known as a multifunctional adaptor protein and is currently recognized for its involvement in tumor malignancy and immune responses [9]. There are two alternatively spliced isoforms of ARRB1 while ARRB1-△exon13 (del exon13) lacks 24 base pairs in exon13 corresponding to ARRB1 [10]. A prior research has indicated that *ARRB1*-△exon13 functions as a translational regulator for GBM cell adaptation to harsh environment [11]. However, the mechanisms by which ARRB1-△exon13 influences GBM growth and invasion remain unexplored. Thus a comprehensive understanding of the functions of ARRB1-△exon13 is warranted.

In the current article, we demonstrate that ARRB1-△exon13 mediates GBM growth and invasion through interactions with glycolytic pathway proteins in both vivo and vitro settings. Specifically, our findings indicate that the overexpression of ARRB1-△exon13 enhances GBM invasion and metastasis to a greater extent compared to the overexpression of ARRB1. Moreover, targeted inhibition of glycolysis was shown to mitigate the cancer-promoting effects of ARRB1-△exon13.

## 2 Materials and methods

### 2.1 Cell culture

U87 and T98G cell lines were obtained from the American Type Culture Collection (ATCC) and were cultured in DMEM (HyClone) supplemented with 10 % fetal bovine serum (FBS) and 1 % penicillin/streptomycin at 37 °C in a humidified atmosphere containing 95 % air and 5 % CO_2_.

### 2.2 Construction of plasmids, transfections and stable cell lines

ARRB1 and ARRB1-△exon13 overexpression lentivirus with the negative control (NC) lentivirus were purchased from Hanyin Biotech (Shanghai, China). Lentiviral particles were produced using the transfer vector (pLVX-M-Puro) with plasmids (pMD2.G and psPAX2). It was done in replicates of three per cell line in addition to one negative transfection control per cell line. Then the resulting supernatant was filtered through a 0.45 μm PVDF filter (VWR), and applied to the GBM cells in the presence of polybrene (5 μg/ml). Six hours post-transfection, fresh complete media was added. Finally, transfected cells were chosen using puromycin for 1 week to establish stable cell lines.

### 2.3 Western blot analysis

Cells were lysed on ice for 30 min using a lysis buffer (Solarbio) containing phenylmethylsulfonyl fluoride (Sigma‒Aldrich) and a protease inhibitor cocktail (Roche). Then cells were centrifugated for 10 min at 12,500 g at a temperature of 4 °C. Subsequently, the samples were separated via SDS‒PAGE and transferred onto PVDF membranes. The membranes were blocked using TBST containing 5 % skim milk and incubated overnight at 4 °C with primary antibodies. HRP-conjugated secondary antibodies were then incubated at room temperature for 1 h and chemiluminescence detection was performed using a ChemiDoc Imaging System (Bio-Rad). The primary antibodies utilized included ARRB1 antibody (Abcam, Cat# ab32099), ALDOA antibody (Abcam, Cat# ab269260), ENO1 antibody (Abcam, Cat# ab227978) and β-actin antibody (Abcam, Cat# ab8226).

### 2.4 CCK8 assay

Following a 24-h culture in 96-well plates, the GBM cells were treated with 10 μL of CCK-8 for a duration of 4 h, after which the absorbance for each well was detected with enzyme standard instrument operating at 450 nm wavelength. A proliferation curve was drawn using the OD values. This experiment was conducted for three times.

### 2.5 Colony formation assay

A 1 ml volume of 0.8 % agar was added to a 6-well plate as the base agar. Subsequently, 500 cells per well (0.5 mL) were combined with 0.5 mL of pre-warmed 20 % FBS-DMEM and an additional 0.5 ml of 0.8 % agar. Once the agar reached a jelly-like consistency, a layer of complete culture medium was added to support cell nourishment. The 24-well plate was then incubated for 14 days. Wells were stained with 0.5 % crystal violet for 1 h, and the colonies were periodically observed using a dissecting microscope. Three biological replicates of cell lines were done.

### 2.6 Transwell assay

Cells (2×10^4^/100 μl) were introduced into the upper chambers of Matrigel-coated Transwell assay inserts (Millipore) and were subsequently placed into the lower chamber of a 24-well plate for 16 h. After that, the top layer of the insert was scrubbed with a sterile cotton swab and the invading cells on the bottom surface were stained with 0.1 % crystal violet for 30 min. The number of cells was then observed and photographed in six independent fields using an inverted microscope. Multiple representative images were taken for quantification of cell migration via Adobe Photoshop's counting tool. Then we opened each image and labeled each cell in the field of view (FOV). The average number of migrated cells per FOV was calculated. This was done in three biological replicates of cell line.

### 2.7 Scratch wound-healing assay

A 200 μl pipette tip was employed to draw three parallel horizontal lines on the underside of a six-well plate, and a straight line perpendicular to the horizontal lines. An appropriate volume of uniformly mixed cells was added to the wells, achieving 100 % confluence after overnight culture. The wells were washed twice with PBS to remove detached cells and serum-free medium was added. Images were captured at 24 and 48 h, and the extent of wound healing was subsequently calculated. The assay was done in replicates of three per cell line.

### 2.8 Quantitative real-time PCR

Cells were lysed using RNAiso Plus (Takara, China, Cat.108-95-2). The RNA was reverse transcribed utilizing an RT reagent kit (Takara, Cat.RR047A). Polymerase chain reaction (PCR) was conducted with FastStart Universal SYBR Green Master Mix (Roche, cat.04194194001) on an Applied Biosystems 7500 machine. The sequences of each forward (F) and reverse (R) primer employed for PCR were ARRB1 (F), AGAGTCTATGTGACGCTGAG and (R), GCACTTGTACTGAGCTGTGTT; GAPDH (F), TGACCTCAACTACATGGTCTACA and (R), CTTCCCATTCTCGGCCTTG.

### 2.9 Co-immunoprecipitation

Whole cell lysates were prepared using NETN lysis buffer (20 mM Tris-pH 8.0, 100 mM NaCl, 1 mM EDTA, and 0.5 % NP-40) supplemented with a protease inhibitor cocktail and phosphatase inhibitors (1 mM sodium orthovanadate and 10 mM NaF). The lysates were subjected to immunoprecipitation with Flag antibody conjugated to agarose beads (Sigma-Aldrich) or MYC and FLAG tag antibodies in conjunction with protein A beads (Millipore) for 2 h at 4 °C with continuous rotation. The immuno-precipitates were subsequently washed with NETN buffer and finally analyzed via western blotting.

### 2.10 Pyruvate measurement

Pyruvate measurement was quantified using a commercially available Pyruvate Assay Kit (Abcam, ab65342). U87 and T98G cells were seeded in six-well plates at a density of 3 × 10^5^ cells per well and incubated at 37 °C for 24 h. Before assays, cells were lysied with lysis buffer provided in the assay kit. Then samples were centrifuged and collected. Finally, pyruvate measurement was assessed according to the manufacturer's instructions, with all outcomes being adjusted based on cell number.

### 2.11 In vivo xenograft model

For the GBM model, four-week-old athymic male nu/nu mice (n = 8 per group) were subjected to one of three different treatment conditions: 1) U87-MG-negative control (NC); 2) U87-MG-ARRB1; and 3) U87-ARRB1-△exon13. The cells (5×10^6^) were injected into the right ganglia region using a small animal stereotactic frame (David Kopf Instruments). Tumor volume was calculated using the formula (volume = length × width^2^/2).

### 2.12 Statistical analysis

The statistical analysis was conducted by SPSS software ver.22.0. Statistical graphs were generated via GraphPad Prism software ver.9.0. One-way ANOVAs, Pearson correlation analyses, and two-tailed Student's t tests were employed to analyze the corresponding data. A P-value of less than 0.05 was deemed statistically significant.

## 3 Results

### 3.1 Alternative splicing of ARRB1-Δexon13 enhances ARRB1 expression in GBM cell lines

**Fig. 1:**
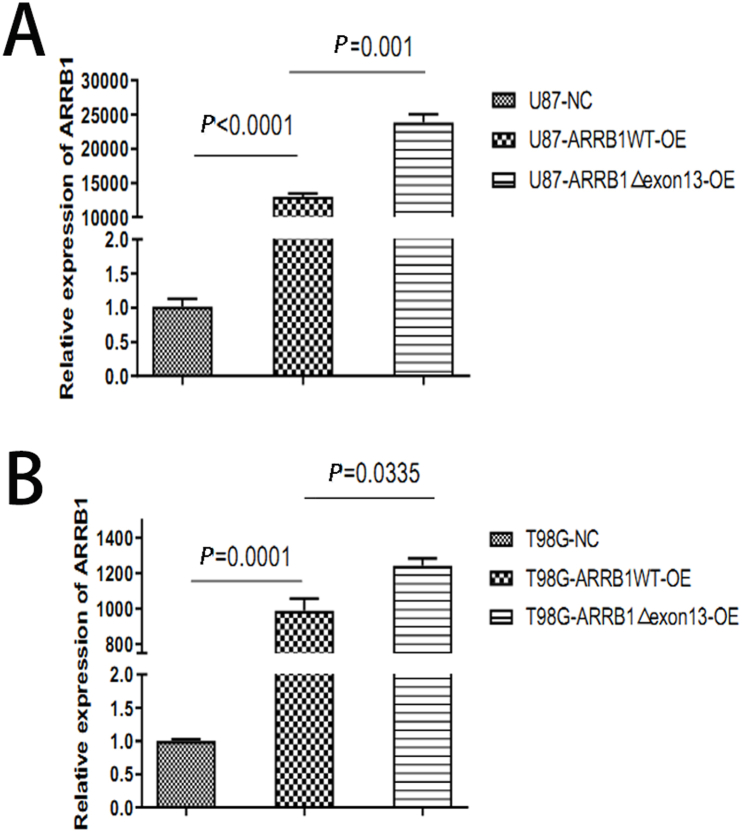
ARRB1 was expressed higher in ARRB1-OE compared to ARRB1-NC GBM cells and the highest in ARRB1 Δexon13-OE GBM cells compared to other 2 GBM cells.(A) RT-PCR assays of the ARRB1 in U87 cells in the following groups: the negative control (NC), and ARRB1-OE, ARRB1-△exon13. (B) RT-PCR assays of the ARRB1 in T98G cells in the groups mentioned in (A). Data were plotted as the mean ± SEM. Statistical analysis was performed using one-way ANOVA followed by Tukey's multiple comparisons test and Dunnett's multiple comparisons test (A,B).

Preliminary published findings have confirmed the existence of exon skipping of ARRB-1 exon13 (ARRB1-Δexon13)within GBM [12]. To further assess the expression levels of ARRB1 in GBM, we constructed lentiviral plasmids to establish ARRB1 normal control (NC), ARRB1-overexpression (OE) and ARRB1Δexon13-OE U87 and T98G cell lines. The expression levels of ARRB1 were evaluated using RT-PCR(Fig. 1). Collectively, results of RT-PCR indicate that ARRB1 expression is significantly higher in ARRB1-OE GBM cells compared to ARRB1-NC GBM cells, with the highest expression observed in ARRB1 Δexon13-OE GBM cells.

### 3.2 Alternative splicing of ARRB1-Δexon13 enhances the proliferation and migration of GBM cell lines

**Fig. 2:**
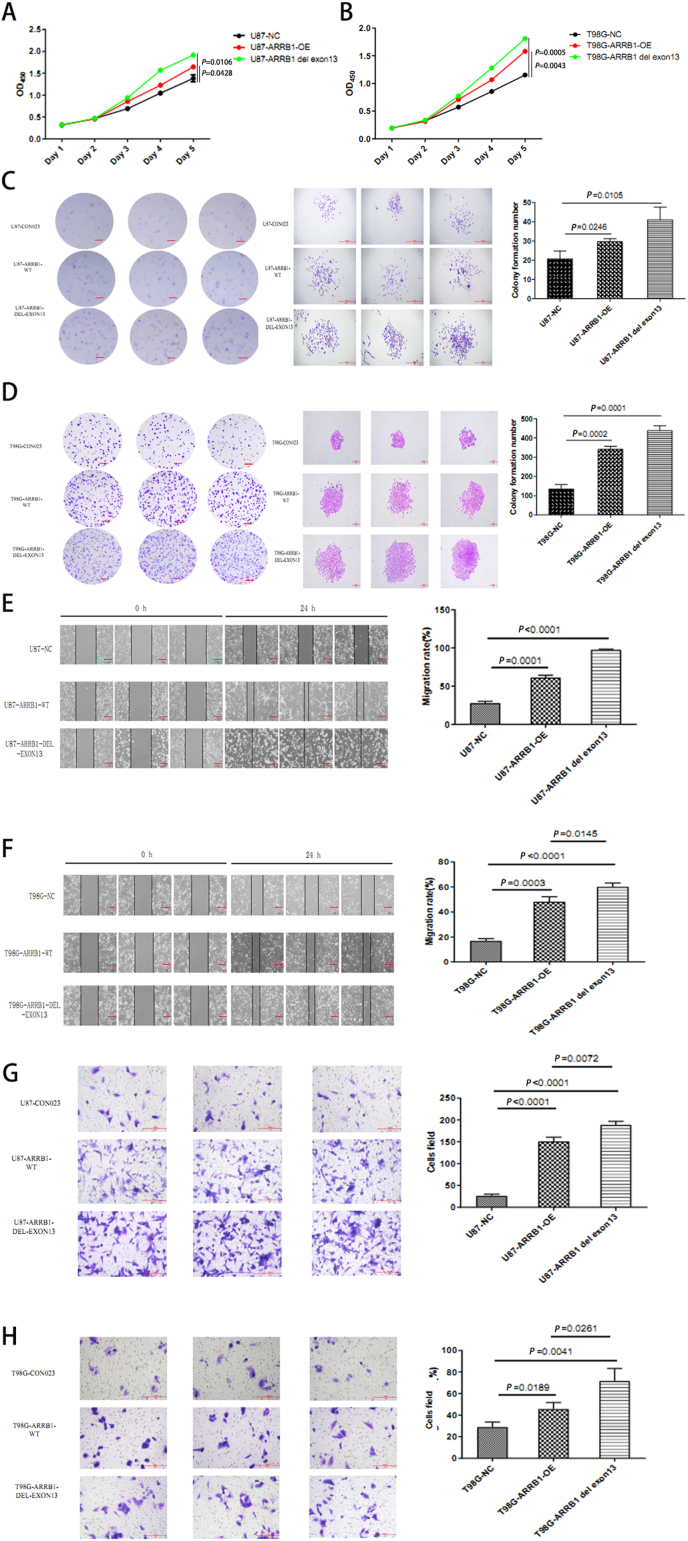
ARRB1-△exon13 proteoform is critical for sustained tumor progression.(A) CCK-8 assays of U87 cells in the following groups: the negative control (NC), ARRB1-OE and ARRB1-△exon13. (B) CCK-8 assays of T98G cells in the groups mentioned in (A). (C) Colony assays of U87 cells in the groups mentioned in (A). Scale bar, 1000um. (D) Colony assays of T98G cells in the groups mentioned in (A). Scale bar, 200um.(E) Cell scratch assays of U87 cells in the groups mentioned in (A). Scale bar, 250um. (F) Cell scratch assays of T98G cells in the groups mentioned in (A). Scale bar, 250um. (G) Transwell assays of U87 cells in the groups mentioned in (A). Scale bar, 200um. (H) Transwell assays of T98G cells in the groups mentioned in (A). Scale bar, 200um.Data were plotted as the mean ± SEM. Statistical analysis was performed using one-way ANOVA followed by Tukey's multiple comparisons test (A, B) and Sidak's multiple comparisons test (C–H).

Emerging evidence suggests that ARRB1 plays a critical role in regulating the self-renewal and growth of cancer cells [13]. To investigate the effects of ARRB1 on GBM cell lines functionality, we conducted proliferation assays, including cell counting kit-8(CCK-8) and colony formation assays. The results demonstrated that the ARRB1-OE isoform enhanced GBM cell proliferation compared to the ARRB1-NC isoform and the ARRB1△exon13-OE isoform further enhanced GBM cell proliferation compared to the ARRB1-OE isoform (Fig. 2A–D). Additionally, migration-related assays, including cell scratch experiment and Transwell assays, indicated that the ARRB1-OE isoform facilitated GBM cell migration, with the ARRB1-△exon13 isoform further augmenting this effect (Fig. 2E–H). Thus, these findings suggest a strong association between ARRB1-△exon13 and GBM proliferation and invasion.

### 3.3 The malignancy-promoting effects of ARRB1-△exon13 can be inhibited by 2-DG

**Fig. 3:**
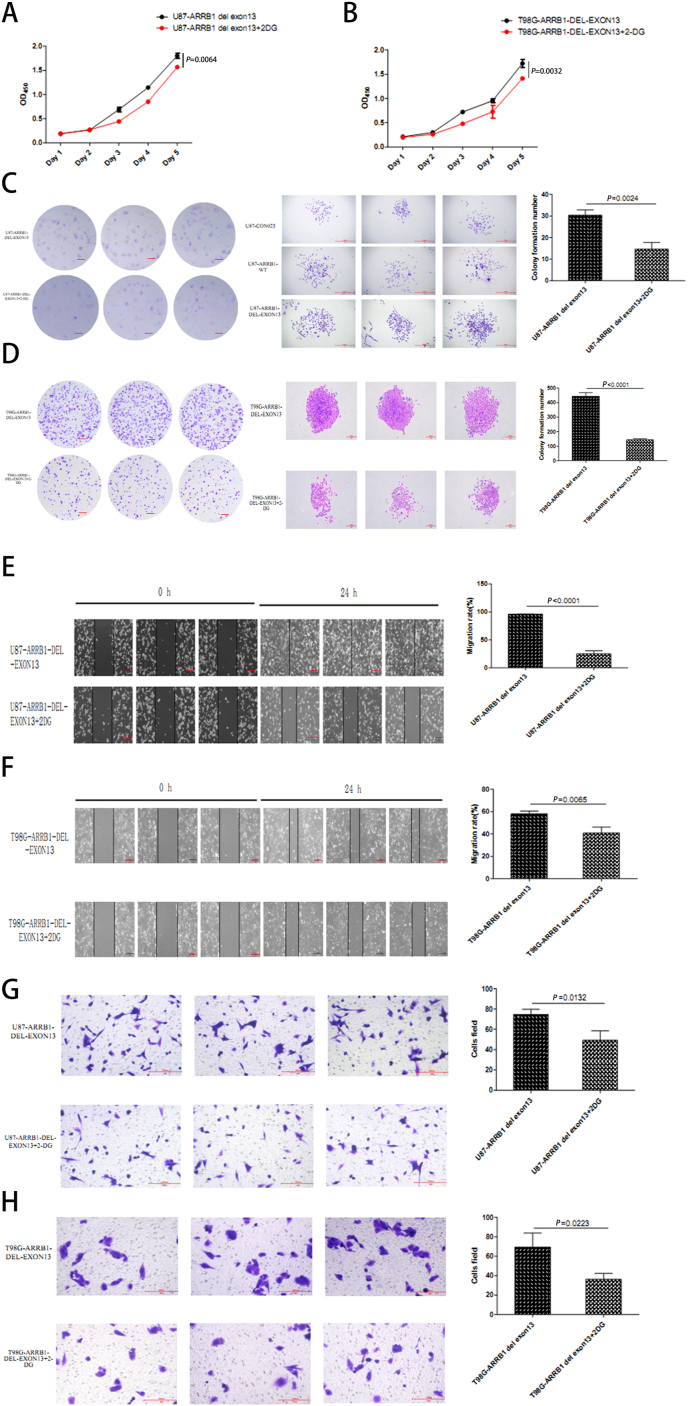
The growth and migration ability of ARRB1-△exon13 could be blocked by 2-DG.(A) CCK-8 assays of U87 cells in the following groups: ARRB1-△exon13 and ARRB1-△exon13+2-DG. (B) CCK-8 assays of T98G cells in the groups mentioned in (A). (C) Colony assays of U87 cells in the groups mentioned in (A). Scale bar, 1000um. (D) Colony assays of T98G cells in the groups mentioned in (A). Scale bar, 200um. (E) Cell scratch assays of U87 cells in the groups mentioned in (A). Scale bar, 250um. (F) Cell scratch assays of T98G cells in the groups mentioned in (A). Scale bar, 250um. (G) Transwell assays of U87 cells in the groups mentioned in (A). Scale bar, 200um. (H) Transwell assays of T98G cells in the groups mentioned in (A). Scale bar, 200um. Data were plotted as the mean ± SEM. Statistical analysis was performed using one-way ANOVA followed by Tukey's multiple comparisons test (A, B) and Sidak's multiple comparisons test (C–H). Data shown were representative of 3 independent experiments.

To elucidate the interaction between ARRB1-△exon13 and the glycolysis pathway, we employed the glycolysis inhibitor 2-Deoxy-d-glucose (2-DG) to inhibit glycolysis in GBM cells and subsequently performed proliferation assays, including CCK-8 and colony formation assays (Fig. 3A–D). The GBM malignancy-promoting capacity of the ARRB1-△exon13 isoform could be partly mitigated by 2-DG, which restricted glucose uptake. Additionally, both cell scratch assays and invasion assays demonstrated that 2-DG effectively inhibited the migratory and invasive abilities of GBM cells expressing ARRB1-△exon13 (Fig. 3E–H). These findings suggest that the malignancy-enhancing effects of ARRB1-△exon13 can be counteracted by the application of a glycolysis inhibitor.

### 3.4 The combination of ARRB1 with glycolysis-related enzymes including aldolase A (ALDOA) and enolase 1 (ENO1)

**Fig. 4:**
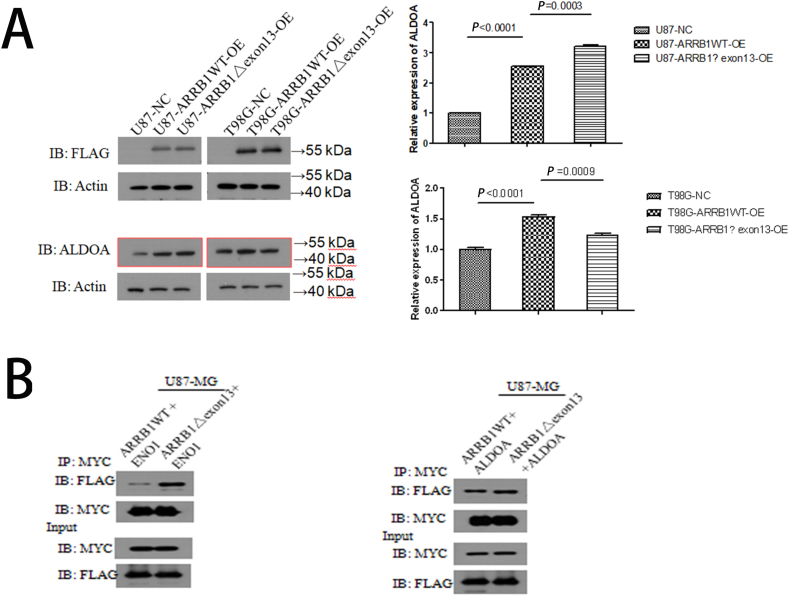
The correlation between ARRB1 and glycolysis-related protein.(A) WB assays of the ARRB1 in U87 and T98G cells in the following groups: the negative control (NC), and ARRB1-OE, ARRB1-△exon13. (B) WB assays of ARRB1-WT and ARRB1-△exon13 with ALDOA and ENO1. (C) Co-immunoprecipitation assays of ARRB1-WT and ARRB1-△exon13 with ALDOA and ENO1.

To elucidate the role of ARRB1 in glycolysis-related pathways, we employed immunoblotting to assess the expression levels of glycolysis-associated enzymes, specifically ALDOA. Our results indicated that over-expression of ARRB1 leads to an increase in ALDOA expression, with the ARRB1-Δexon13 isoform further up-regulating this expression (Fig. 4A). Western blot (WB) and Co-immunoprecipitation (CO-IP) experiments were conducted to investigate the interaction between ARRB1-Δexon13 and glycolysis-related proteins, revealing that ARRB1-Δexon13 enhanced the expression of enzymes such as ALDOA and ENO1 compared to ARRB1 WT (Fig. 4B and C).

### 3.5 ARRB1-Δexon13 isoform increases pyruvic acid content

**Fig. 5:**
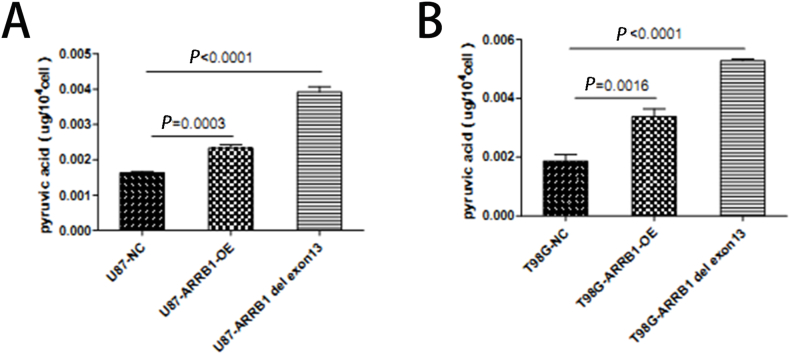
ARRB1-△exon13 splicing was associated with high pyruvic acid content in GBM. (A) Pyruvic acid in the following groups of U87 cells: the negative control (NC), ARRB1-OE, and ARRB1-△exon13. (B) Pyruvic acid in the following groups of T98G cells; the negative control (NC), ARRB1-OE, and ARRB1-△exon13. Statistical analysis was performed using one-way ANOVA followed by Sidak's multiple comparisons test. Data shown were representative of 3 independent experiments.

Furthermore, recent studies have highlighted the significance of pyruvic acid as a critical energy source for tumor cell proliferation and metastasis [14]. Our findings demonstrated a significant increase in pyruvate acid levels following ARRB1 overexpression compared to the ARRB1-NC isoform, with the ARRB1Δexon13-OE isoform exhibiting an even greater increase in GBM cells relative to the ARRB1-OE isoform (Fig. 5).

### 3.6 In vivo experiments reveal that the ARRB1-△exon13 isoform promotes tumor growth

**Fig. 6:**
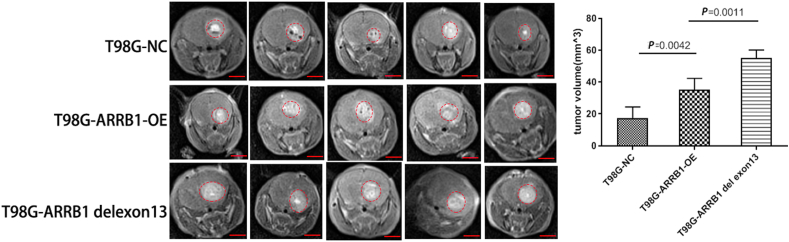
ARRB1 and ARRB1-△exon13 isoform promotes tumor growth in vivo. (A,B) Tumor volume in mice intracranial GBM from the NC, ARRB1-OE and ARRB1-△exon13 groups (n = 5). The area of the tumor is circled in red. The tumor volume was calculated in each group by the Coniglobus formula method. Scale bar, 4 mm. Statistical analysis was performed using unpaired *t*-test. Data shown were representative of 3 independent experiments.

Mice were monitored daily, and tumor diameters were assessed via MRI. After one month, GBMs were isolated from brain tissues, and the result indicated that the ARRB1-△exon13 isoform significantly enhanced tumor growth compared to the ARRB1-NC and ARRB1-OE isoforms (Fig. 6).

Collectively, these data suggested that ARRB1 promotes the hyperplasia and infestation of GBM, with the ARRB1-△exon13 isoform intensifying these effects. The glycolysis inhibitor 2-DG could inhibit the malignancy-promotion capabilities of both ARRB1 and ARRB1-△exon13, indicating that ARRB1 is involved in glycolysis pathways. Furthermore, the interaction of ARRB1 with glycolysis-related proteins, including ENO1 and ALDOA, has been substantiated.

## 4 Discussion

GBM is recognized as one of the most aggressive tumors in humans; however, research on the relationship between alternative splicing (AS) and splicing factors in GBM remains limited [15]. AS events were found to be applied in over 95 % of transcribed human genes by Walter Gilbert in 1978 [16]. These AS events and the resultant isoforms are closely linked to cancer malignancy and poor prognosis and thus may be potential biomarkers or therapeutic targets for cancers [17]. In this study, we verified the ARRB1 del exon13 (△exon 13) isoform as a product of AS events in GBM, and found ARRB1-△exon 13 isoform was associated with cancerous proliferation, metastasis and metabolic reprogramming.

In recent years, the study of the Warburg Effect from oxidative phosphorylation to aerobic glycolysis has made a breakthrough in various tumors including colorectal cancers and pancreatic cancers [18]. β-arrestin1 (ARRB1) was initially known as an adaptor protein involved in G protein-coupled receptor desensitization [19]. Additionally, ARRB1 has been implicated in tumor vascularization, metastasis and metabolic reprogramming [20]. Notably, our prior research indicated that ARRB1 enhanced cancer cell growth through its association with glycolysis and the hypoxia-inducible factor 1 (HIF-1) signaling pathway [21]. The current study elucidates a new molecular mechanism by which ARRB1 mediates GBM proliferation and migration. Furthermore, the ARRB1-△exon13 isoform was verified to enhance GBM proliferation and migration, correlating with glycolysis-related proteins including ALDOA and ENO1.

An increasing body of literature has identified glycolysis-related proteins as critical targets of anti-tumor therapies [22]. Specifically, the glycolytic enzymes ALDOA and ENO1 have been found to be highly expressed in GBM [23]. In this investigation, we demonstrated that the expression level of ALDOA and ENO1 was significantly higher in ARRB1-OE GBM cells than in ARRB1-NC GBM cells and the highest in ARRB1△exon13-OE GBM cells. AlDOA (Aldolase, Fructose-Bisphosphate A) catalyzes the conversion of fructose-1,6-bisphosphate to glyceraldehyde 3-phosphate (G3P) and dihydroxyacetone phosphate (DHAP) which is closely related to the Warburg Effect of tumor metabolism [24]. ENO1 catalyzes the transition of 2-phosphoglyceric acid to phosphoenolpyruvic acid during glycolysis [25]. ENO1 functions as a multifunctional oncoprotein contributing to seven of the ten hallmarks of cancer [26]. Additionally, ENO1 exhibits moonlighting functions on the cell surface and acts as a plasminogen receptor, promoting cancer hyperplasia and metastasis by inducing angiogenesis [27]. In the present study, we have confirmed the interaction between ARRB1 with ALDOA and ENO1 via co-immunoprecipitation (CO-IP), although the precise mechanism underlying these interactions warrants further analysis.

The function of 2-deoxy-d-glucose (2-DG) to induce glucose restriction has been utilized in cancer treatment [28]. Nevertheless, accumulating evidence suggests that 2-DG alone is ineffective in eradicating tumors, and its impact on changing tumor radio-responses also remains contentious [29]. In our research, we demonstrated that the proliferation and invasion-promoting capabilities of ARRB1-△exon13 in GBM cells could be partially inhibited by 2-DG, attributed to its role in limiting glucose uptake [30].

Previous studies have indicated that pyruvic acid is an intermediate compound in the energy metabolism affected by glycolysis-related enzymes including aldolase A (ALDOA) and enolase 1 (ENO1) and is involved in both aerobic and anaerobic metabolism [31]. As malignant neoplasms, GBM cells depend on the conversion of pyruvate to lactate to regenerate NADH into NAD^+^ and sustain metabolic flux through glycolysis. Although this process is energetically less favorable than oxidative phosphorylation, it enables GBM cells to survive in unfamiliar tissue microenvironments and facilitates their metastasis within the brain.

To the best of our knowledge, this study represents the first investigation into the role of ARRB1-△exon13 in regulating GBM proliferation and migration through its influence on glycolysis. Collectively, these findings indicate that targeting ARRB1-△exon13 may constitute a significant anti-tumor strategy, particularly for GBMs that depend on glycolysis for energy, thereby allowing for the simultaneous targeting of cancer metabolism. This approach could enhance the efficacy of conventional cancer treatments aimed at inducing cancer cell death. However, further research is necessary to evaluate the viability of cancer-targeting strategies that inhibit the expression of ARRB1-△exon13. It is important to acknowledge certain limitations in our study. Primarily, the results were derived solely from in vivo and in vitro experiments investigating the functions and molecular mechanisms of ARRB1-△exon13 in GBM progression, which would benefit from corroboration through clinical studies. Additionally, our in-vitro experiment utilized MRI scans of a xenograft tumor mouse model, indicating a need for further in-vitro investigations to elucidate additional functions of ARRB1-△exon13 in GBM.

Overall, this study conducted a thorough examination of ARRB1 expression in GBM cell lines, specifically in the contexts of NC, ARRB1-OE and ARRB1-△exon13. We also identified that the isoform ARRB1-△exon13 binds to glycolysis-related enzymes, including ALDOA and ENO1. Furthermore, ARRB1-△exon13 could enhance glycolysis in GBM cells by interacting with these glycolysis-related proteins and increase pyruvic acid as the energy of GBM cell lines needed for the growth and invasiveness, thereby promoting GBM cell proliferation and migration. Notably, these findings provide valuable insights suggesting that targeting the del exon13 proteoforms may serve as potential diagnostic and therapeutic targets.

## Data availability

The original dates presented in the study are included in the Supplementary Material. Further inquiries can be directed to the corresponding authors.

## Ethics statement

Ethics approval was obtained from the ethics committee of Pudong Hospital, School of Medicine, Fudan University. Mice were operated on and housed according to the criteria outlined in the Guide for the Care and Use of Laboratory Animals. Animal experimental procedures were approved by the Institutional Animal Care and Use Committee of Fudan University (IRB number, 2022–13).

## Author contributions

Wei Zi-Long: Conceptualization, Methodology, Data curation. Han Shuo: Conceptualization, Methodology, Writing-original draft. Han Dong Hua: Conceptualization, Methodology, Writing-review and editing. Li Xue-Tao: Conceptualization, Methodology, Formal analysis. Huang Yu-Lun: Validation, Supervision. Wang Zhi-Min: Supervision, Fund provision.

## Funding

This research was supported by the Suzhou Science and Technology Plan Project (Nos. SZM2022004) and the Science and Technology Development Fund of Shanghai Pudong New Area (KPKJ2022-Y104).

## Declaration of competing interest

The authors declare that the research was conducted without any commercial relationships that could be perceived as a potential conflict of interest.

## Data Availability

Data will be made available on request.
