# Supplementary figures and images for: β-suppressor protein 1 (ARRB1)-△exon13 modulates the progression of glioblastoma via combination with glycolysis-related proteins
Source: Biochem Biophys Rep. 2025 May 13;42:102048. doi: 10.1016/j.bbrep.2025.102048 (PMC12142532; doi:10.1016/j.bbrep.2025.102048)

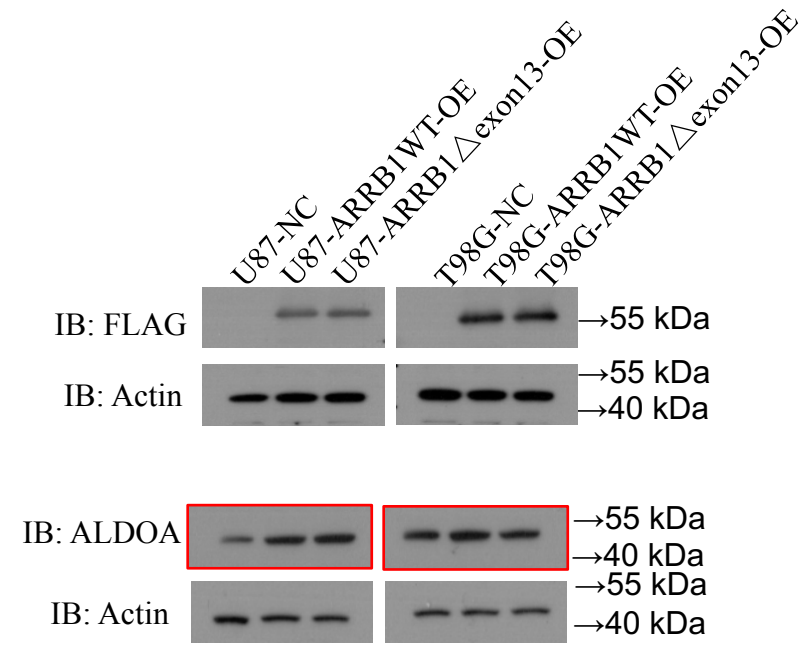

ALDOA

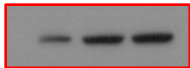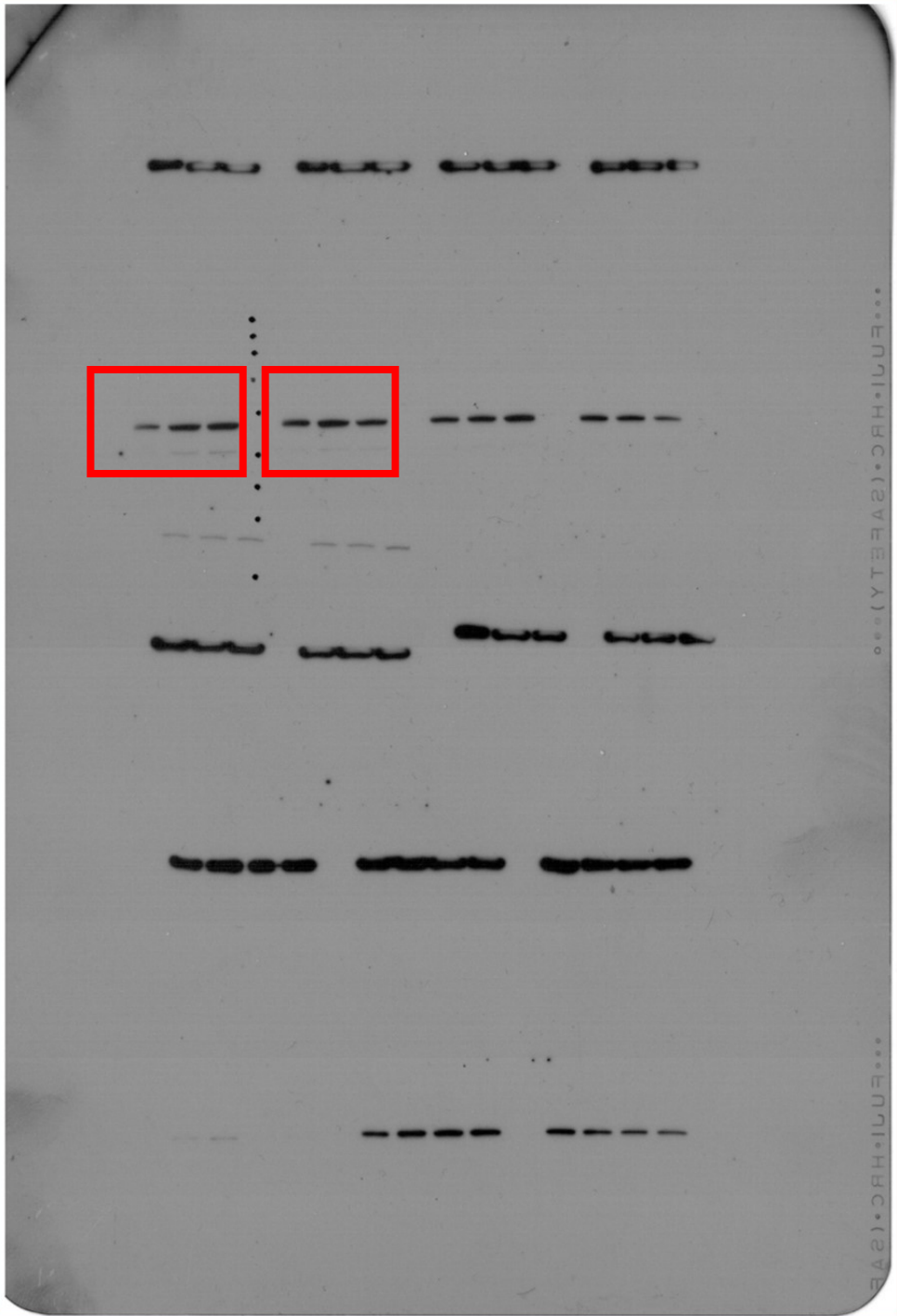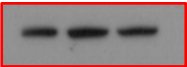

ACTIN

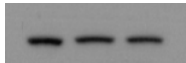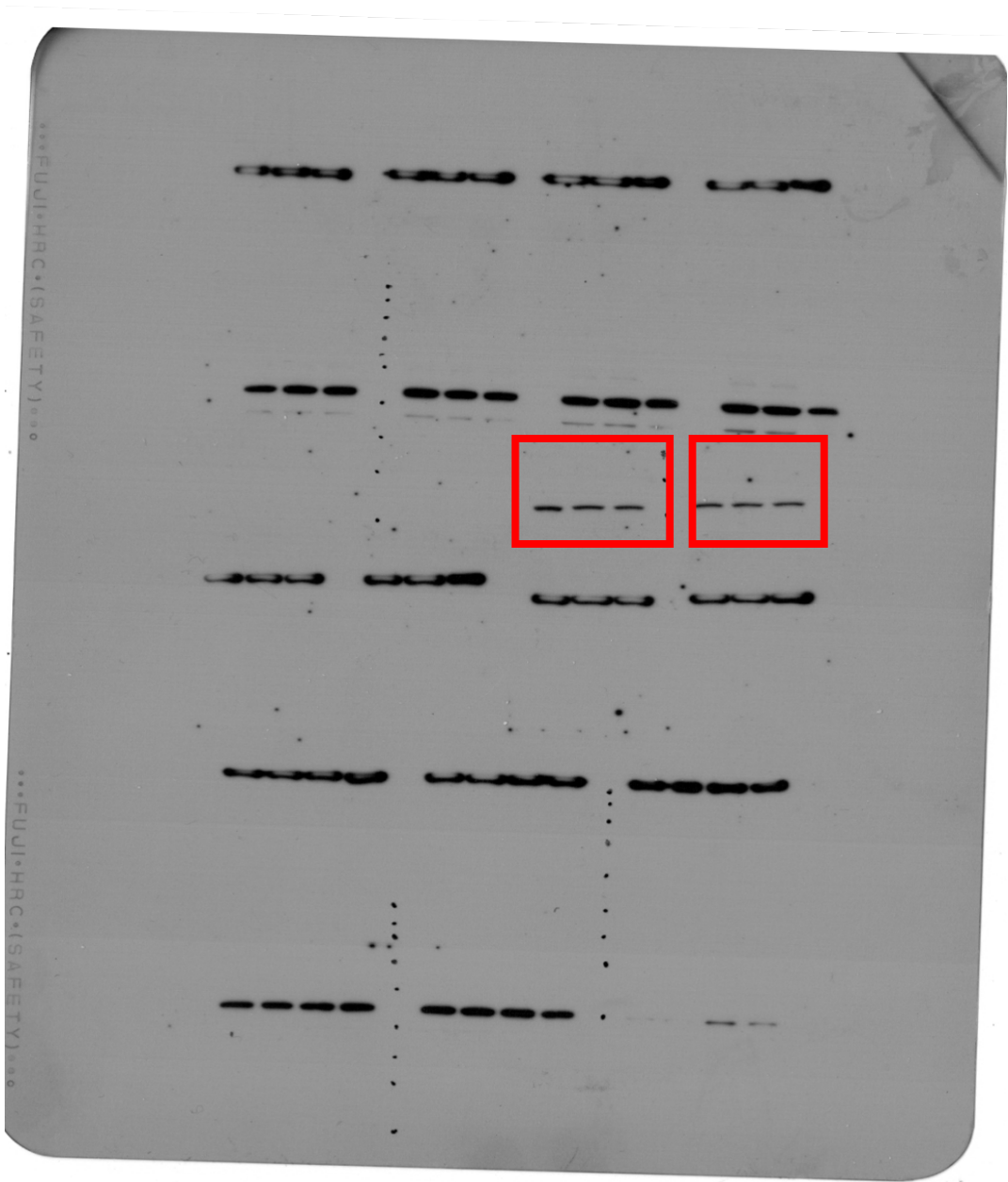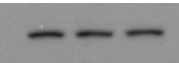

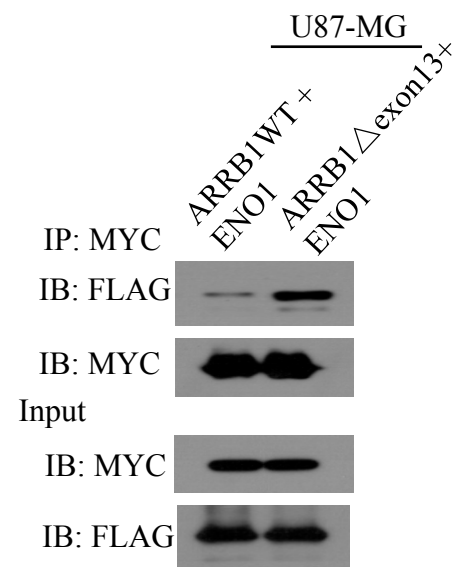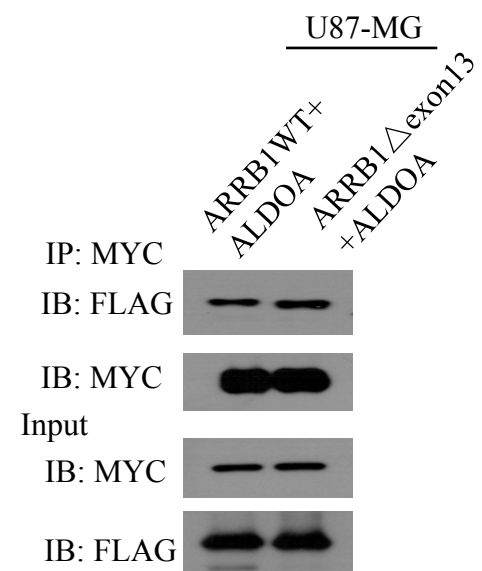

IP FLAG

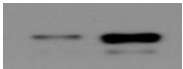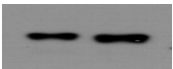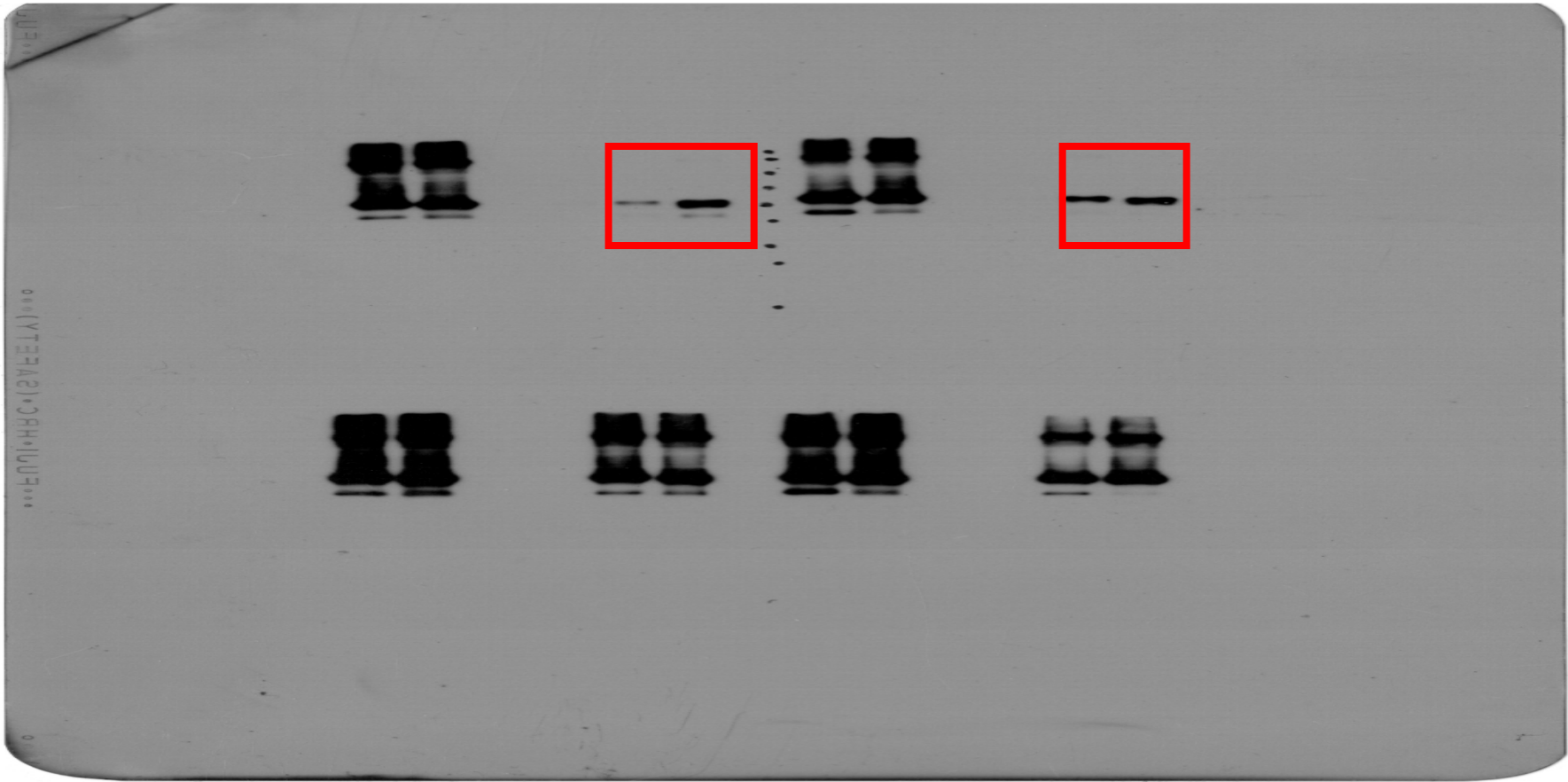

IP MYC

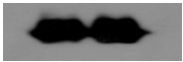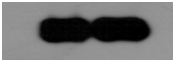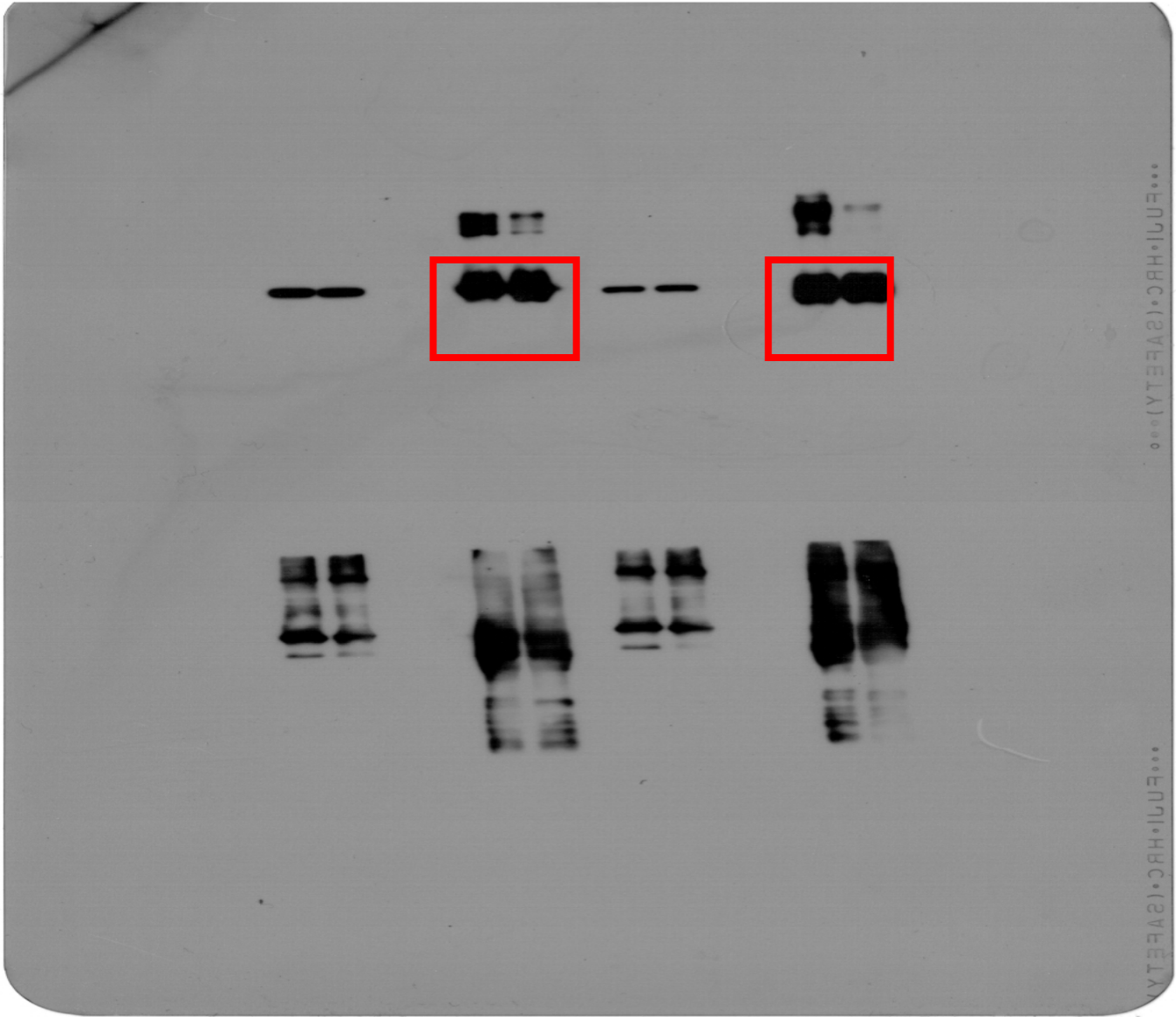

MYC INPUT

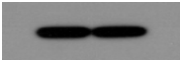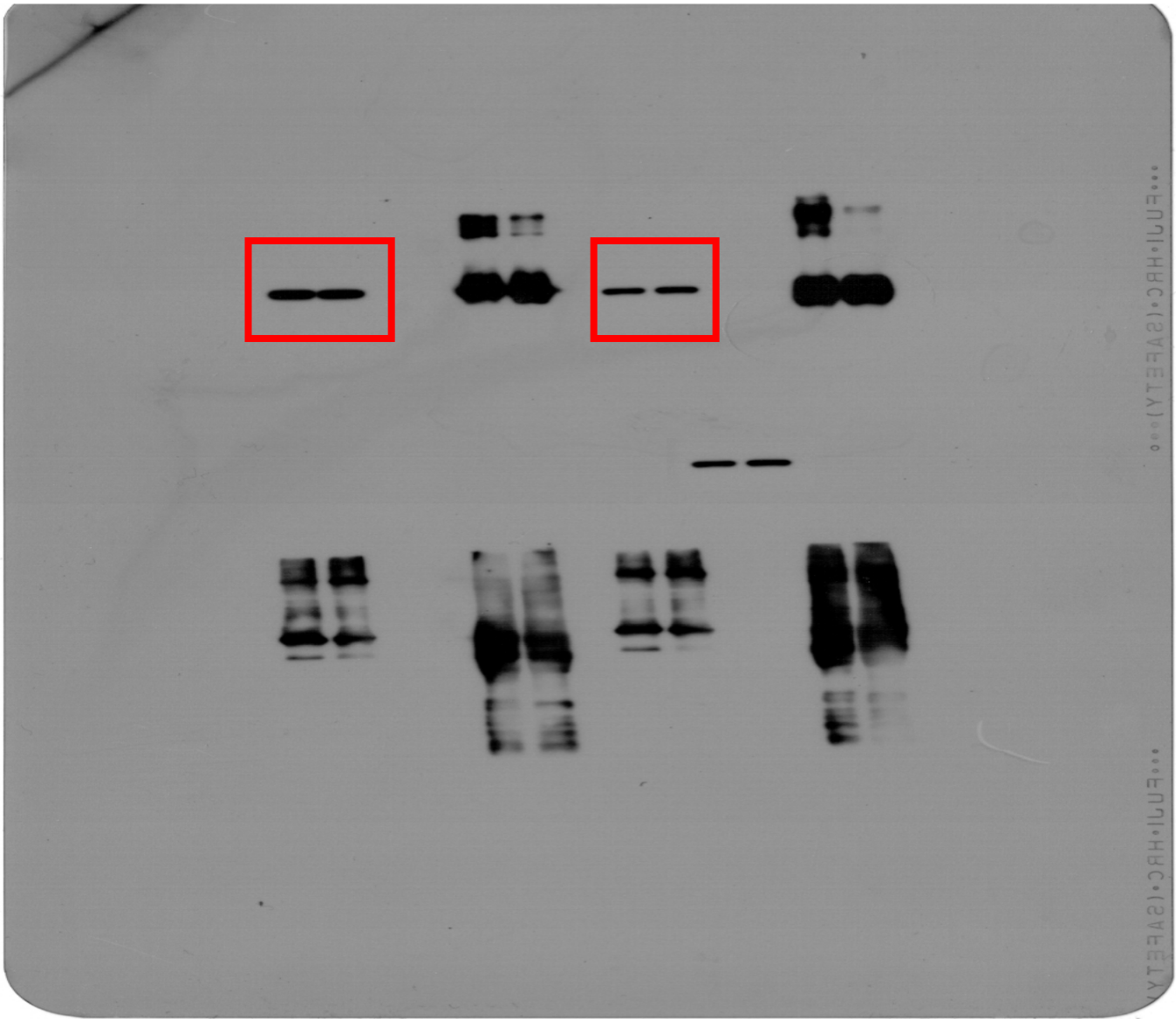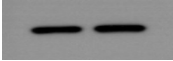

flag INPUT

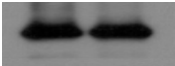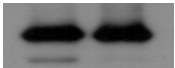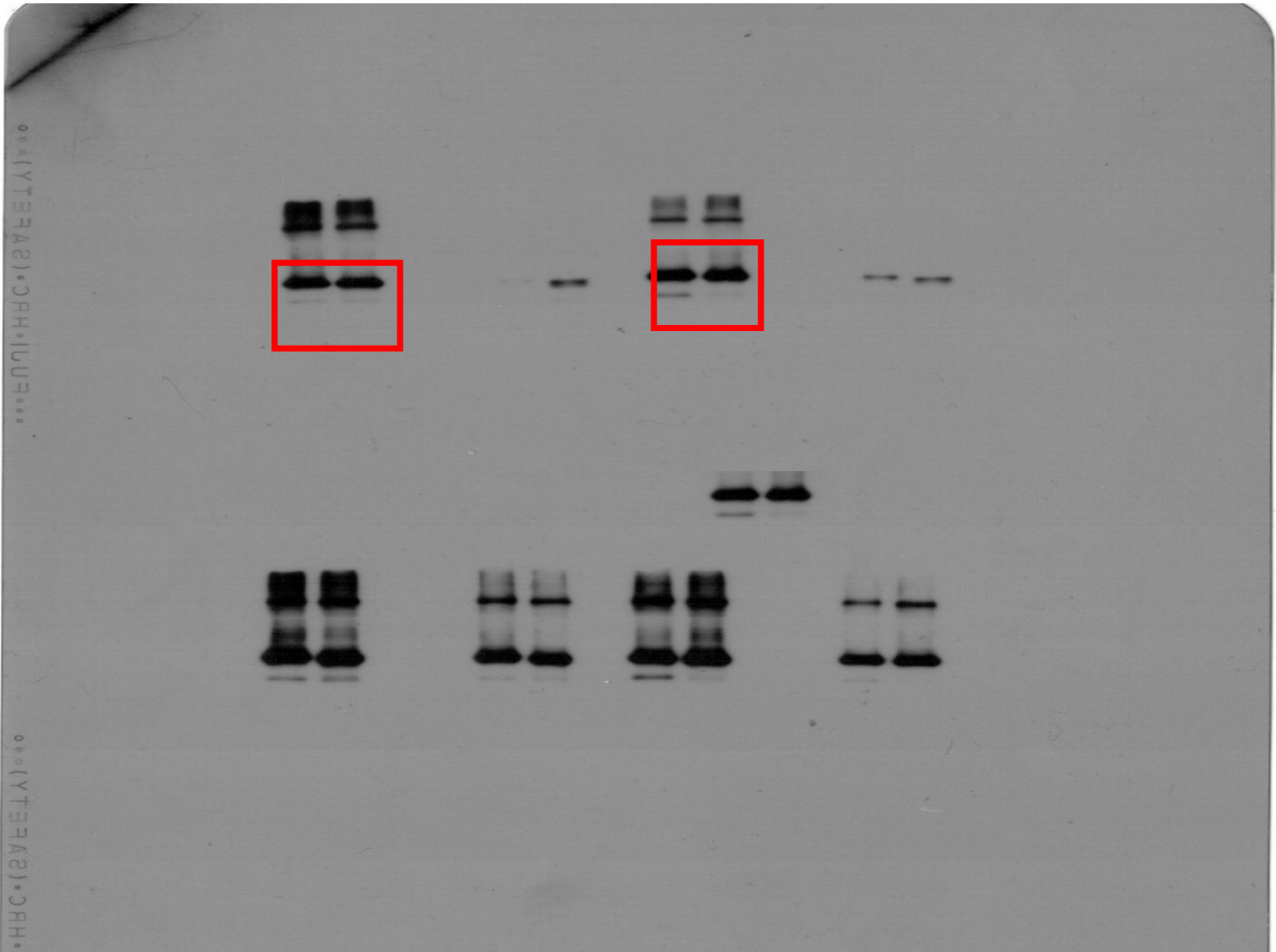

Supplement: Multimedia component 1 [file mmc1.pdf]
