# Supplementary material for: β-suppressor protein 1 (ARRB1)-△exon13 modulates the progression of glioblastoma via combination with glycolysis-related proteins
Source: Biochem Biophys Rep. 2025 May 13;42:102048. doi: 10.1016/j.bbrep.2025.102048 (PMC12142532; doi:10.1016/j.bbrep.2025.102048)
Supplement: Multimedia component 2 [file mmc2.pdf]

1

RT-qPCR

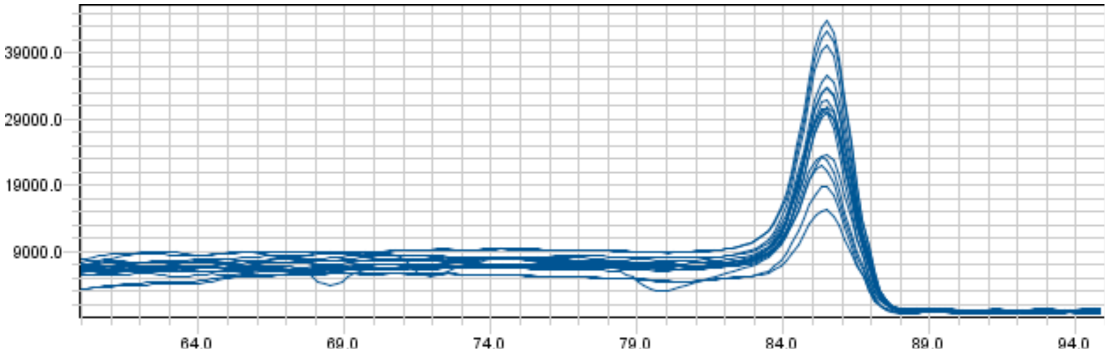

Human ARRB1

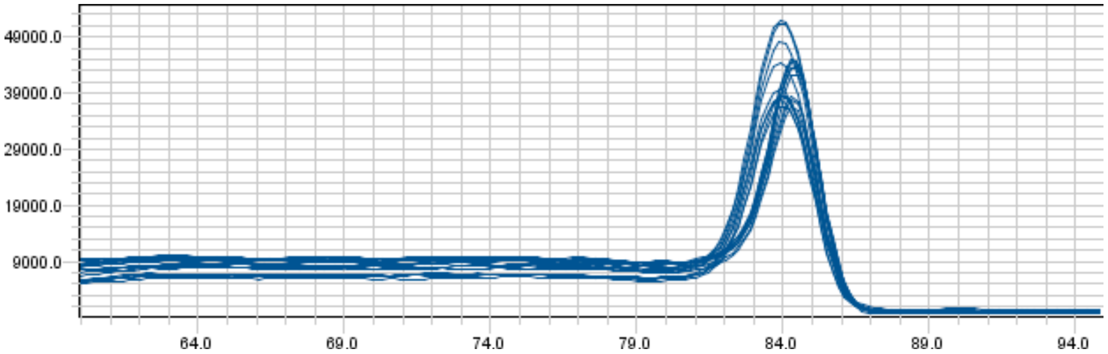

Human GAPDH

	Ct	Ct	mean Ct	detla Ct	mean detla Ct	detla detla Ct	mean detla detla Ct	2- detla detla Ct	mean 2- detla detla Ct		P vaule
	Human ARRB1	Human GAPDH	Human GAPDH	Human ARRB1	Human ARRB1	Human ARRB1	Human ARRB1	Human ARRB1	Human ARRB1	SD	
**T98G-NC**	28.033	14.067	14.2556	13.7777	13.7767	0.0010	0.0000	0.9993	1.0006	0.0416	
	27.972	14.255		13.7162		-0.0605		1.0428			
	28.092	14.445		13.8363		0.0595		0.9596			
**T98G-ARRB1WT-OE**	17.993	14.168	14.1539	3.8394	3.8338	-9.9373	-9.9430	980.4574	988.9823	118.1142	0.0001
	17.813	14.094		3.6589		-10.1178		1111.1280			
	18.157	14.199		4.0030		-9.7737		875.3615			
**T98G-ARRB1△exon13-OE**	18.009	14.450	14.4134	3.5957	3.4992	-10.1810	-10.2775	1160.9077	1242.6290	71.6139	0.0335
	17.852	14.305		3.4386		-10.3381		1294.4341			
	17.877	14.486		3.4632		-10.3135		1272.5451			

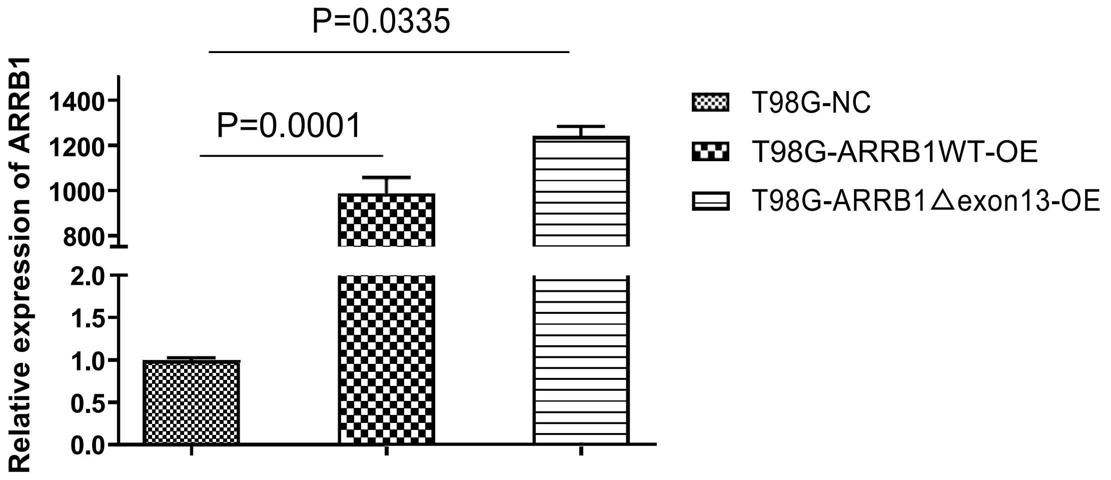

P=0.0335
P=0.0001
Relative expression of ARRB1
1400
1200
1000
800
2.0
1.5
1.0
0.5
0.0
T98G-NC
T98G-ARRB1WT-OE
T98G-ARRB1△exon13-OE

	Ct	Ct	mean Ct	detla Ct	mean detla Ct	detla detla Ct	mean detla detla Ct	2- detla detla Ct	mean 2- detla detla Ct		P value
	Human ARRB1	Human GAPDH	Human GAPDH	Human ARRB1	Human ARRB1	Human ARRB1	Human ARRB1	Human ARRB1	Human ARRB1	SD	
**U87-NC**	31.312	14.443	14.4430	16.8694	16.8608	0.0086	0.0000	0.9941	1.0131	0.2002	
	31.585	14.503		17.1416		0.2808		0.8231			
	31.014	14.383		16.5714		-0.2894		1.2221			
**U87-ARRB1WT-OE**	16.996	13.507	13.8007	3.1953	3.1936	-13.6655	-13.6672	12993.5722	13024.1897	778.3154	0.0000
	16.907	13.888		3.1066		-13.7542		13817.3619			
	17.080	14.007		3.2789		-13.5819		12261.6348			
**U87-ARRB1∆exon13-OE**	17.610	15.199	15.2838	2.3263	2.3217	-14.5345	-14.5391	23731.0351	23864.8060	2027.6280	0.0010
	17.481	15.310		2.1970		-14.6638		25956.0072			
	17.725	15.342		2.4417		-14.4191		21907.3757			

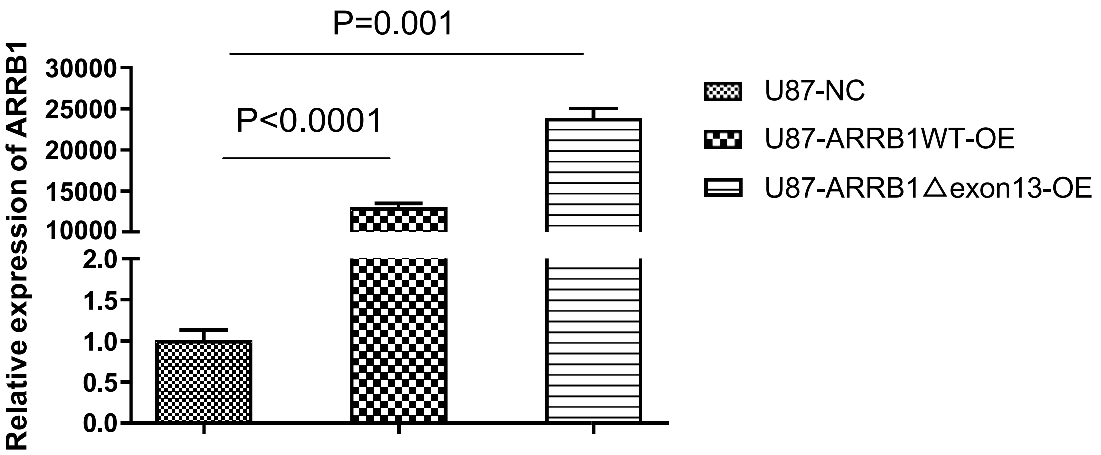

P=0.001
P<0.0001
Relative expression of ARRB1
30000
25000
20000
15000
10000
2.0
1.5
1.0
0.5
0.0
U87-NC
U87-ARRB1WT-OE
U87-ARRB1△exon13-OE

2

Western blot

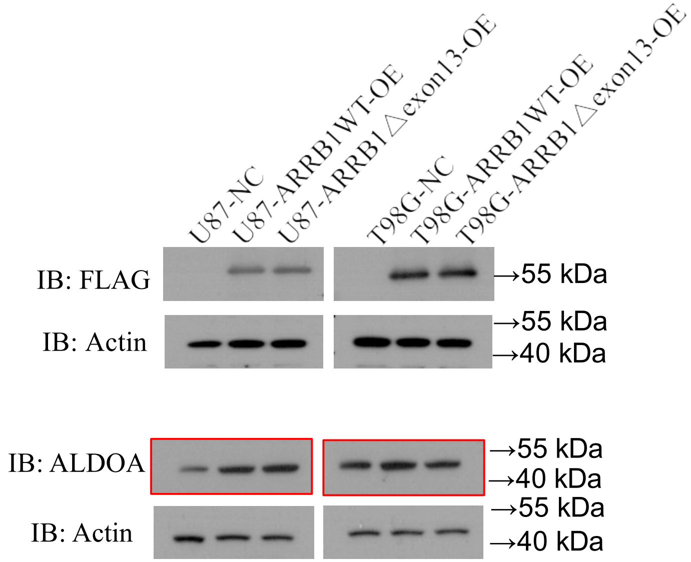

U87-NC
U87-ARRB1WT-OE
U87-ARRB1△exon13-OE
T98G-NC
T98G-ARRB1WT-OE
T98G-ARRB1△exon13-OE
IB: FLAG
→55 kDa
IB: Actin
→55 kDa
→40 kDa
IB: ALDOA
→55 kDa
→40 kDa
IB: Actin
→55 kDa
→40 kDa

3 Co-immunoprecipitation

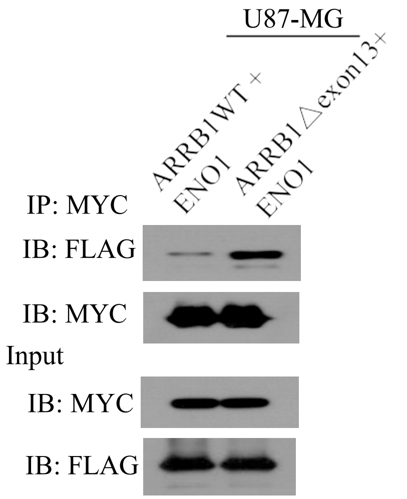
U87-MG
ARRB1WT+ ENO1
ARRB1△exon13+ ENO1
IP: MYC
IB: FLAG
IB: MYC
Input
IB: MYC
IB: FLAG

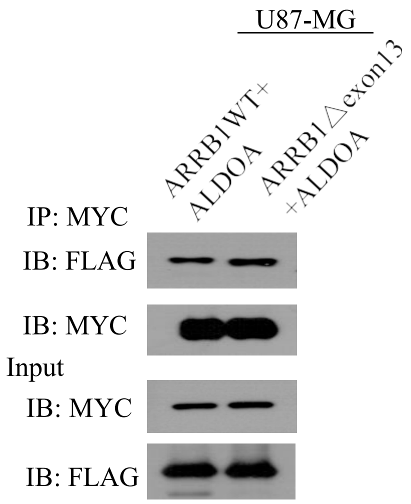
U87-MG
ARRB1WT+ ALDOA
ARRB1△exon13 +ALDOA
IP: MYC
IB: FLAG
IB: MYC
Input
IB: MYC
IB: FLAG

4

CCK8

	T98-NC			T98G-ARRB1-WT			T98G-ARRB1-DEL-EXON13		
Day 1	0.1911	0.1973	0.1929	0.1990	0.1951	0.1921	0.1996	0.1976	0.1933
Day 2	0.3389	0.3195	0.3252	0.3084	0.3227	0.3131	0.3391	0.3345	0.3372
Day 3	0.5906	0.5609	0.5641	0.7081	0.6915	0.7275	0.7825	0.7441	0.7962
Day 4	0.8938	0.8460	0.8253	1.0643	1.0630	1.0756	1.2955	1.2458	1.3025
Day 5	1.1384	1.1918	1.1263	1.5951	1.5819	1.5637	1.8157	1.8065	1.8084

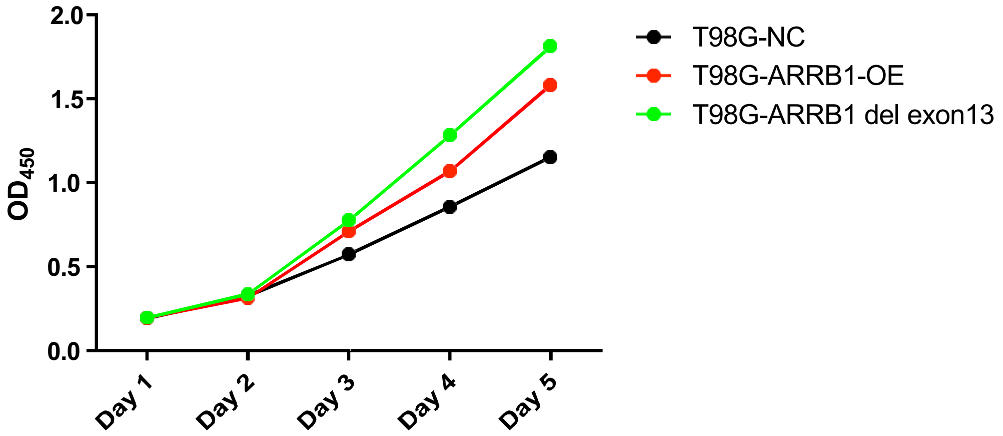

	U87-NC			U87-ARRB1-WT			U87-ARRB1-DEL-EXON13		
Day 1	0.3185	0.3150	0.3215	0.3237	0.3309	0.3257	0.3375	0.3203	0.3134
Day 2	0.4684	0.4656	0.4609	0.4553	0.4523	0.4564	0.4481	0.4805	0.4722
Day 3	0.6828	0.6754	0.7192	0.8653	0.8507	0.8563	0.9213	0.9827	0.9216
Day 4	1.0622	1.0258	1.0521	1.2038	1.2247	1.2561	1.5680	1.5509	1.5982
Day 5	1.3507	1.3233	1.4828	1.6674	1.6170	1.6559	1.9581	1.8843	1.9170

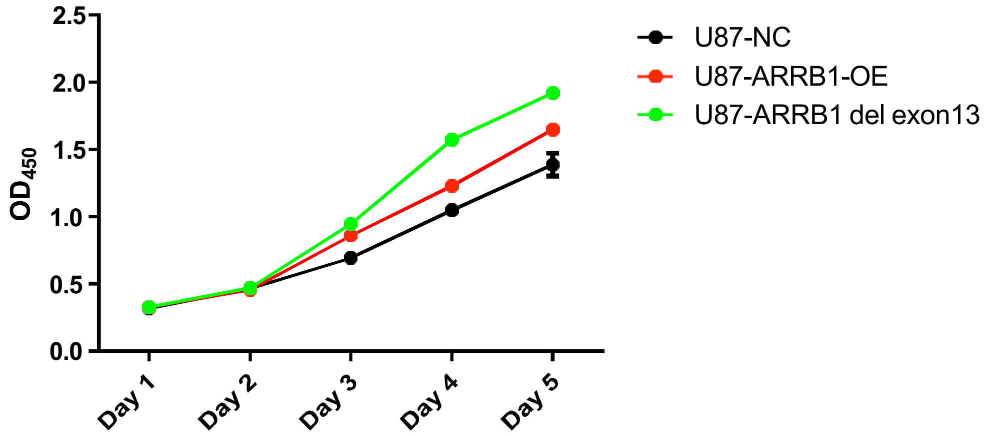

	T98G-ARRB1-DEL-EXON13			T98G-ARRB1-DEL-EXON13+2-DG		
Day 1	0.2177	0.212	0.2005	0.2118	0.1914	0.1904
Day 2	0.3170	0.3068	0.2712	0.2634	0.2598	0.2603
Day 3	0.7467	0.7402	0.6821	0.4665	0.4795	0.4891
Day 4	0.9559	0.9961	0.9127	0.628	0.6728	0.876
Day 5	1.79982	1.7361	1.6362	1.4287	1.4097	1.4038

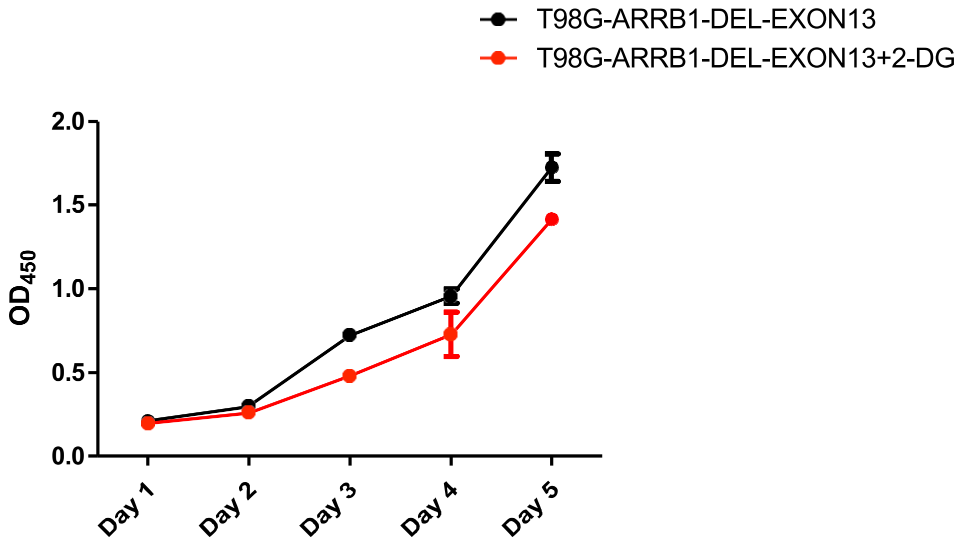

	U87-ARRB1-DEL-EXON13			U87-ARRB1-DEL-EXON13+2-DG		
Day 1	0.1908	0.191	0.1939	0.1959	0.1817	0.1874
Day 2	0.2728	0.2377	0.2978	0.2823	0.2680	0.2652
Day 3	0.7319	0.7021	0.6365	0.4305	0.4692	0.4352
Day 4	1.1758	1.1092	1.1523	0.8653	0.8286	0.8642
Day 5	1.7925	1.7579	1.8611	1.527	1.5912	1.5891

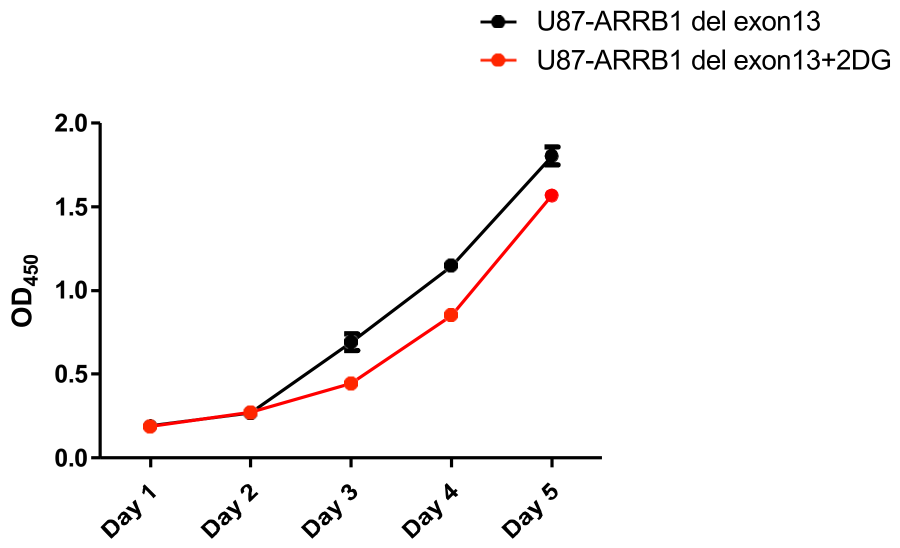

5 Cell Scratch

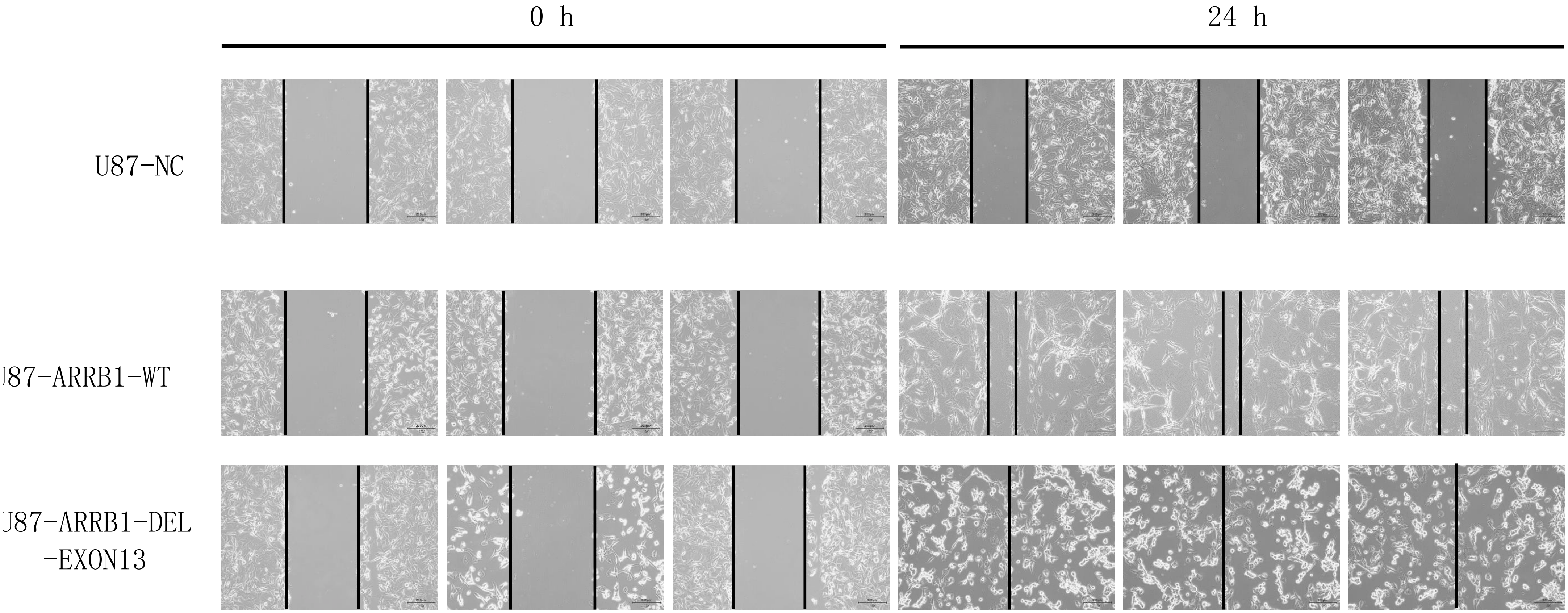
0 h
24 h
U87-NC
J87-ARRB1-WT
J87-ARRB1-DEL
-EXON13

	migration rate（%）			SD	P value
U87-NC	25.83	27.03	30.26	2.2943	
U87-ARRB1-OE	58.33	64.29	61.32	2.9762	0.0001
U87-ARRB1-del exon13	96.95	98.29	97.69	0.6712	0.0000

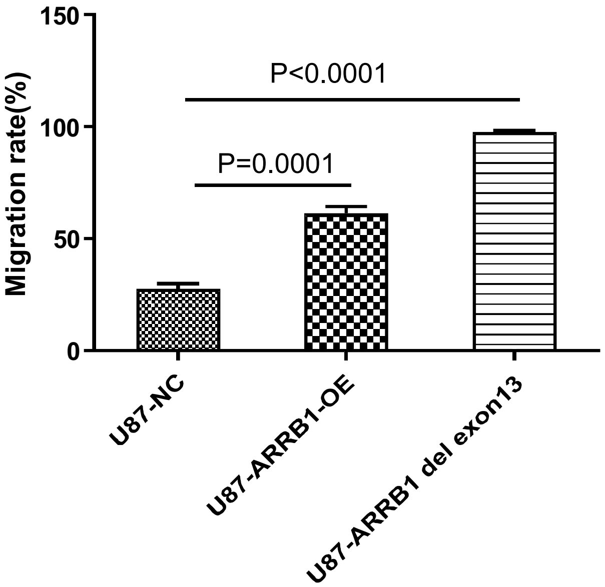

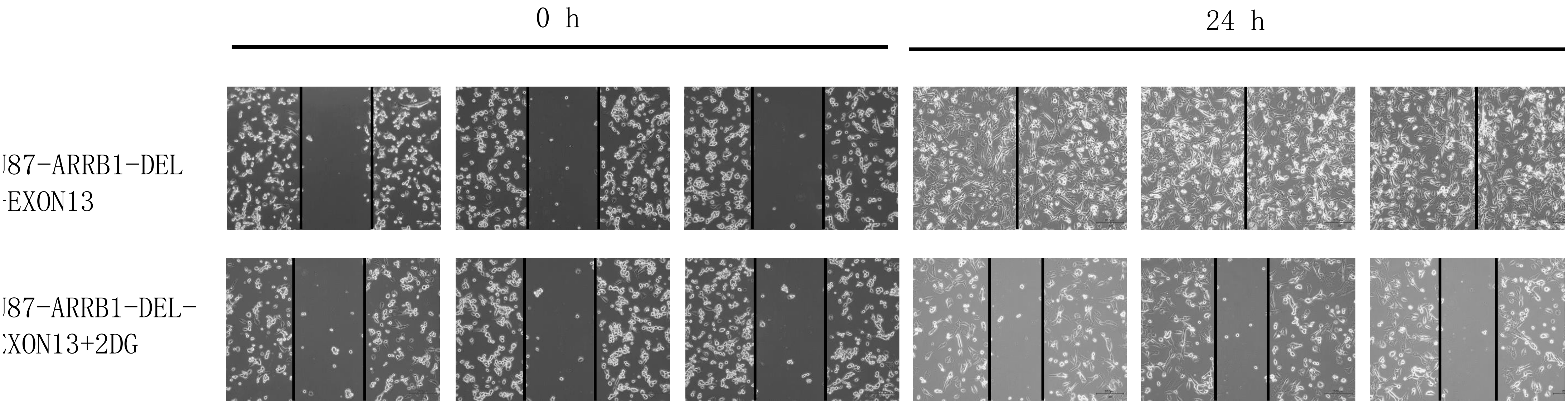
0 h
24 h
87-ARRB1-DEL
EXON13
87-ARRB1-DEL-
XON13+2DG

	migration rate（%）			SD	P value
U87-ARRB1-del exon13	95.45	95.62	96.57	5.5348	
U87-ARRB1-del exon13+2DG	19.01	26.32	29.87	0.6036	0.0000

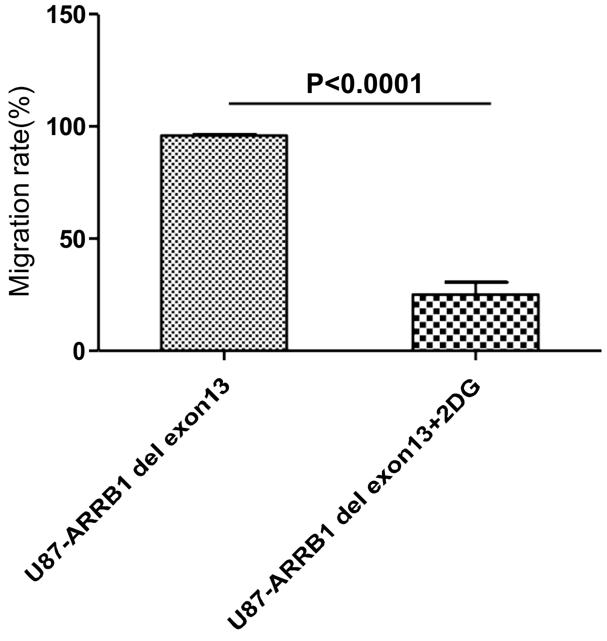

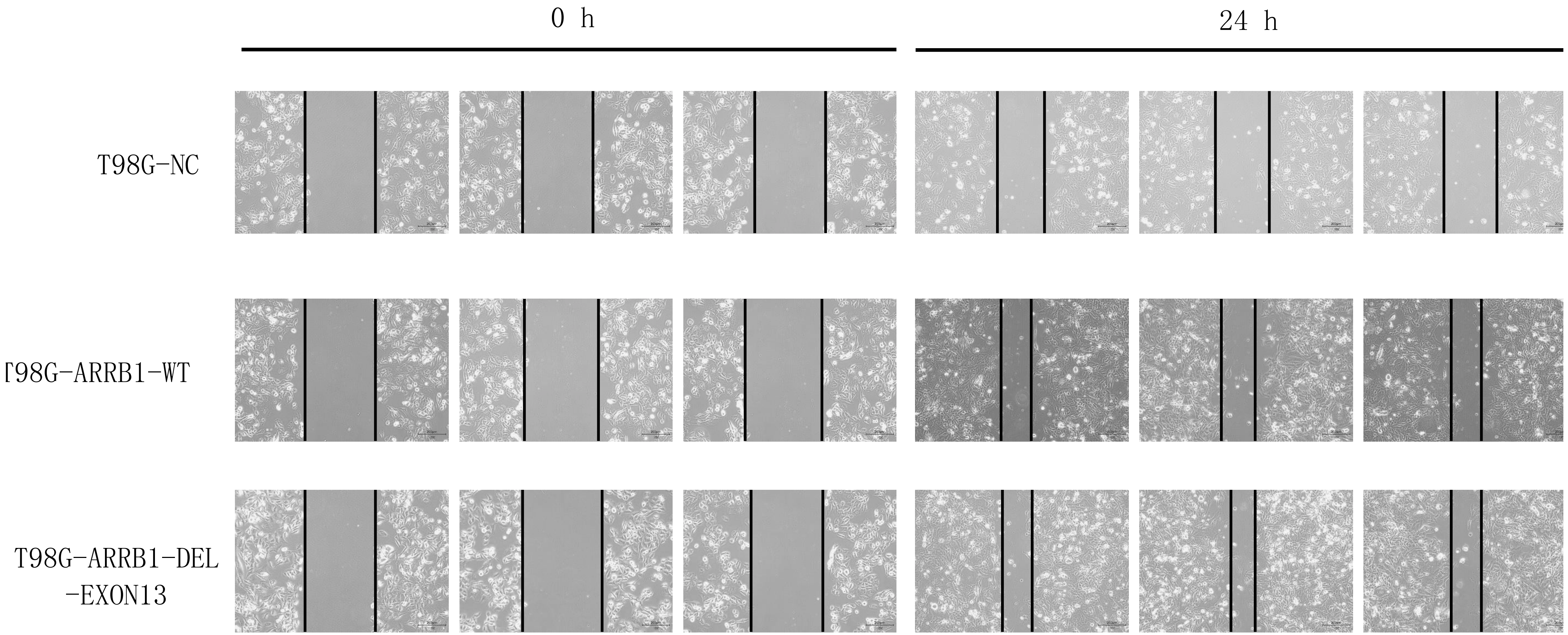
0 h
24 h
T98G-NC
T98G-ARRB1-WT
T98G-ARRB1-DEL
-EXON13

	migration rate（%）			SD	P value
T98G-NC	14.81	17.19	18.60	1.9149	
T98G-ARRB1-OE	51.85	43.75	48.84	4.0949	0.0003
T98G-ARRB1-del exon13	59.26	57.81	63.49	2.9494	0.0000

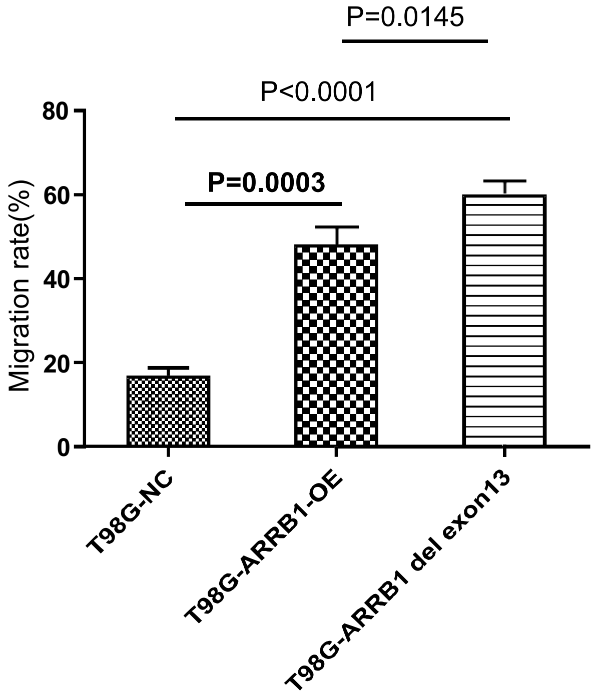

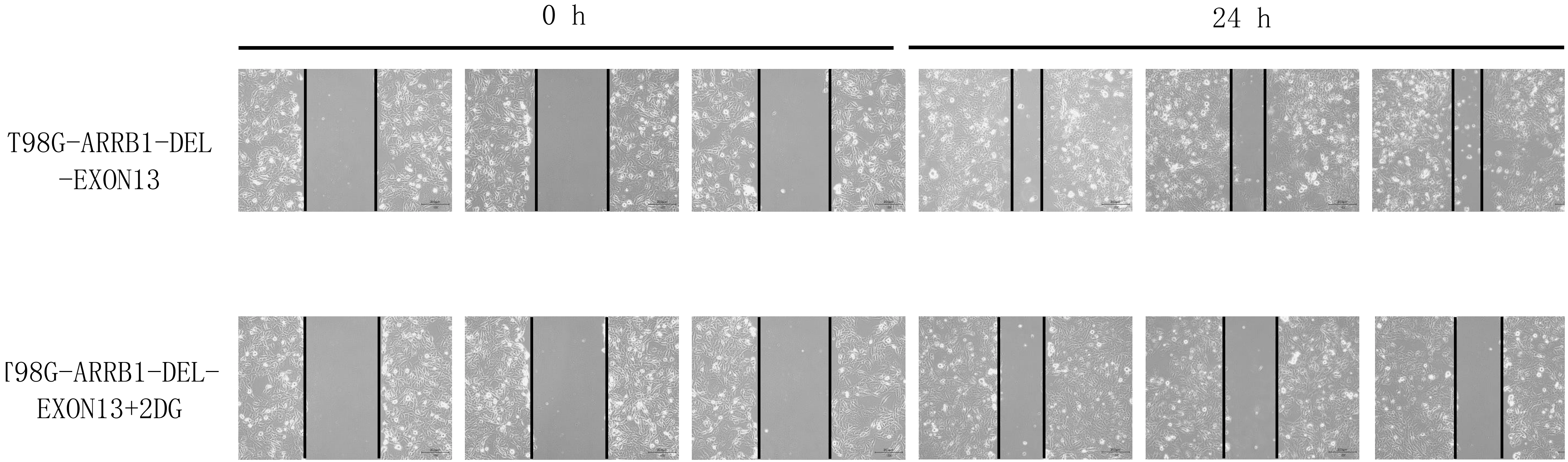
0 h
24 h
T98G-ARRB1-DEL
-EXON13
T98G-ARRB1-DEL-
EXON13+2DG

	migration rate（%）			SD	P value
T98G-ARRB1-del exon13	58.62	55.71	60.28	5.1997	
T98G-ARRB1-del exon13+2DG	41.38	35.71	46.10	2.3127	0.0064

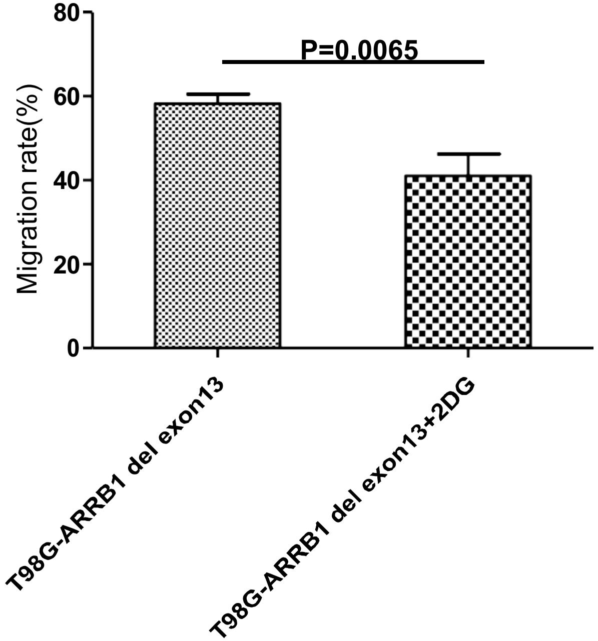

6 Cell Invasion

U87-CON023

U87-ARRB1-
WT

U87-ARRB1-
DEL-EXON13

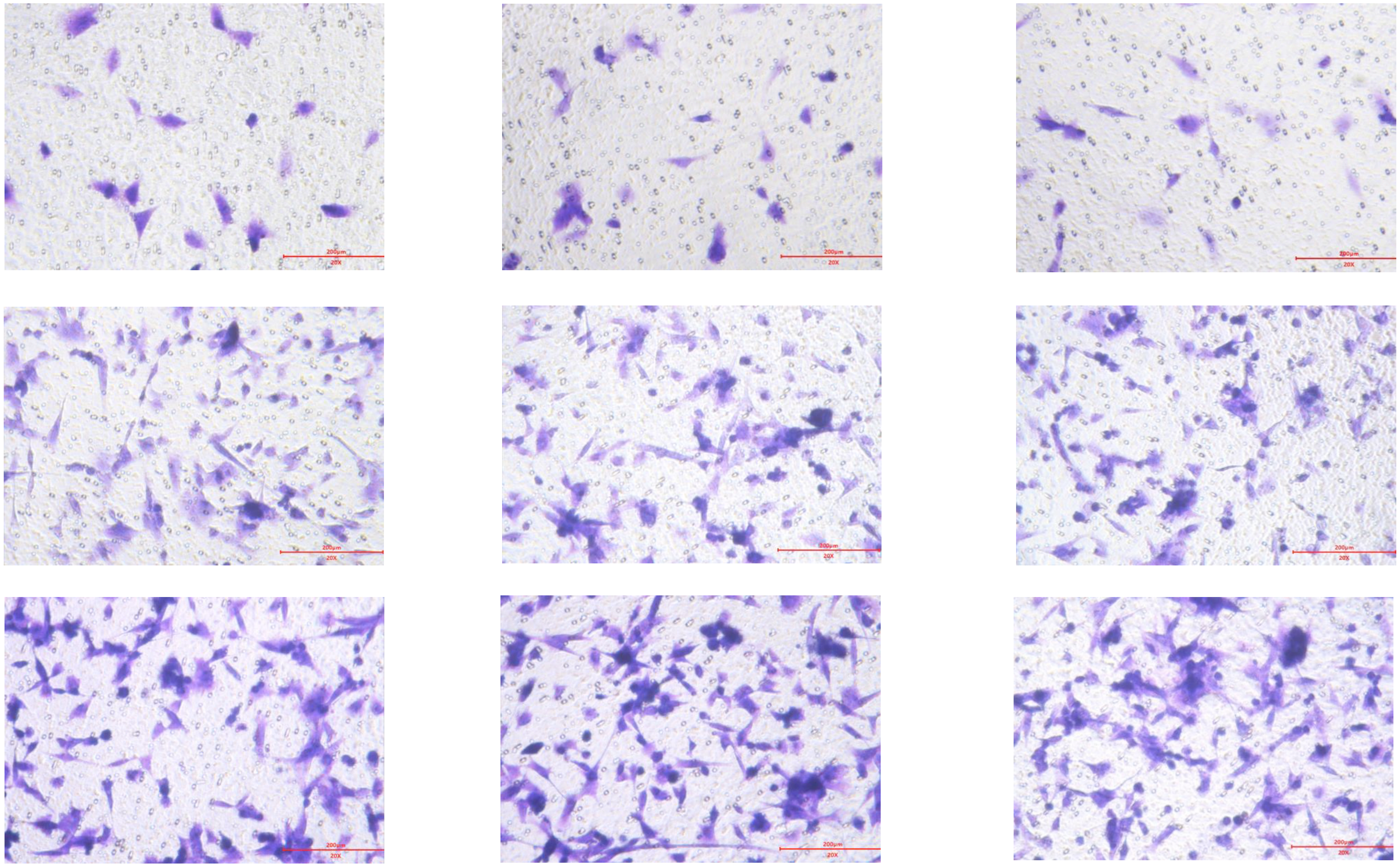

	**Cells/field**			SD	P value
U87-NC	22	30	26	4.0000	
U87-ARRB1-OE	150	142	161	9.5394	0.0000
U87-ARRB1-del exon13	185	198	182	8.5049	0.0000

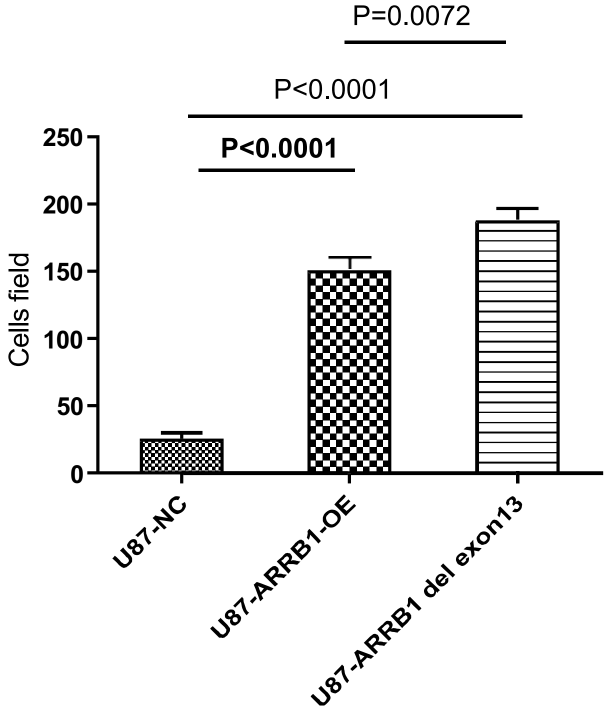

T98G-CON023

T98G-ARRB1-WT

T98G-ARRB1-DEL-EXON13

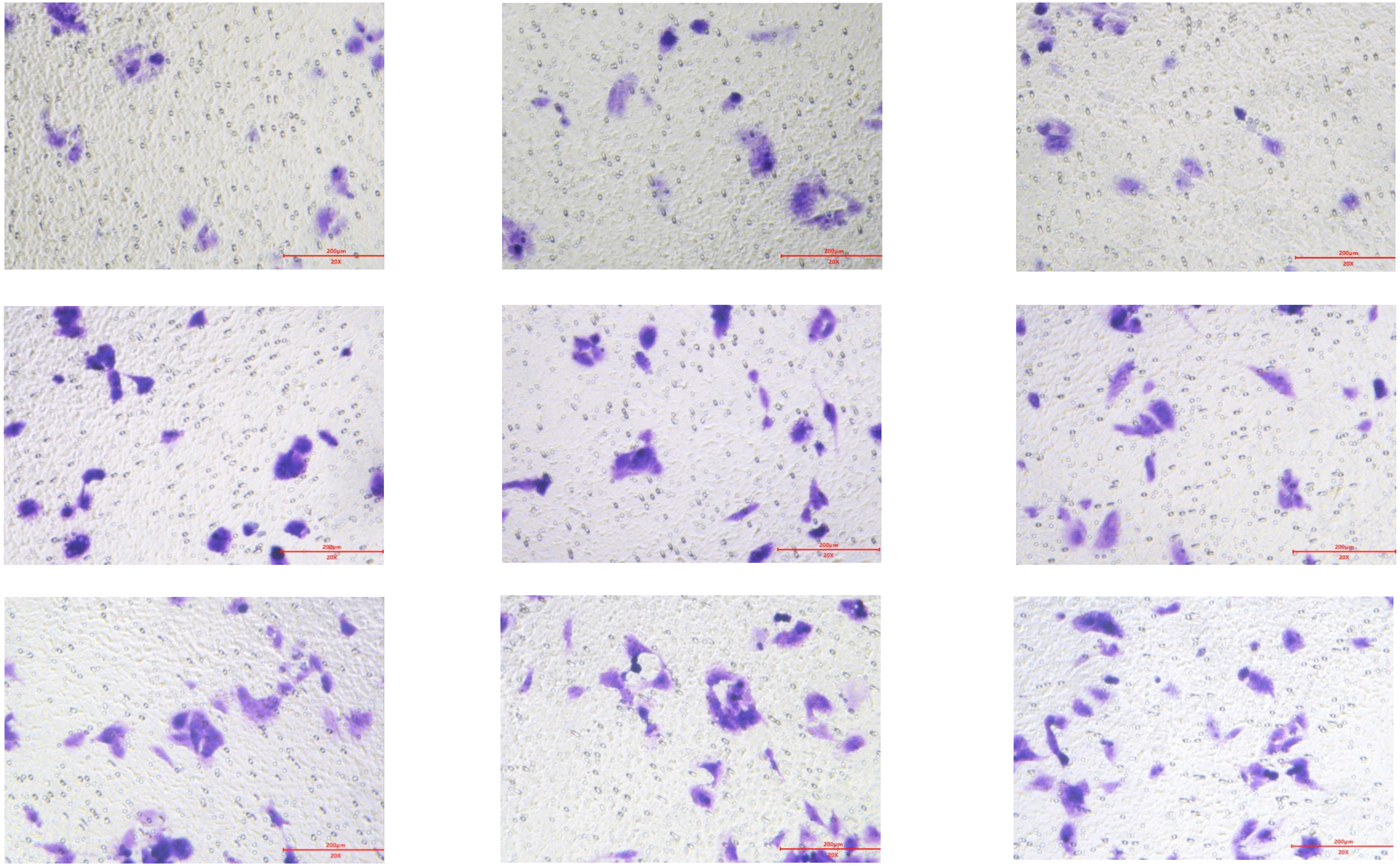

	**Cells/field**			SD	P value
T98G-NC	28	34	25	4.5826	
T98G-ARRB1-OE	45	40	52	6.0277	0.0189
T98G-ARRB1-del exon13	61	70	84	11.5902	0.0041

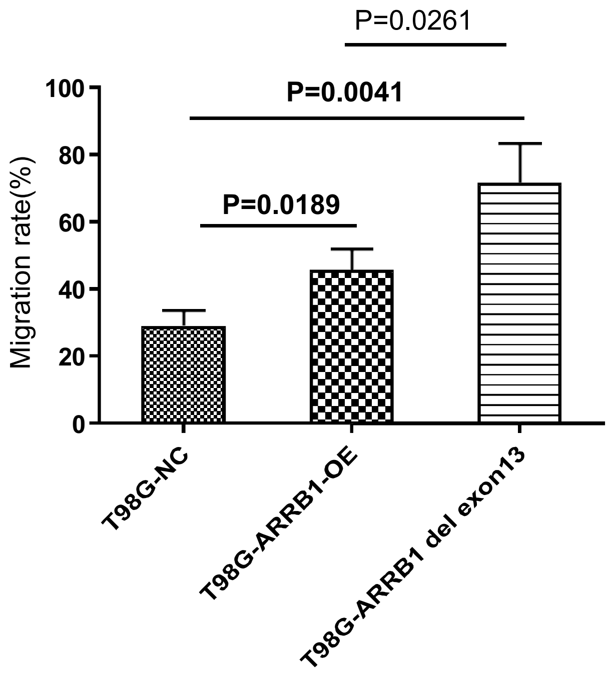

U87-ARRB1-
DEL-EXON13

[illegible]87-ARRB1-DEL-
EXON13+2-DG

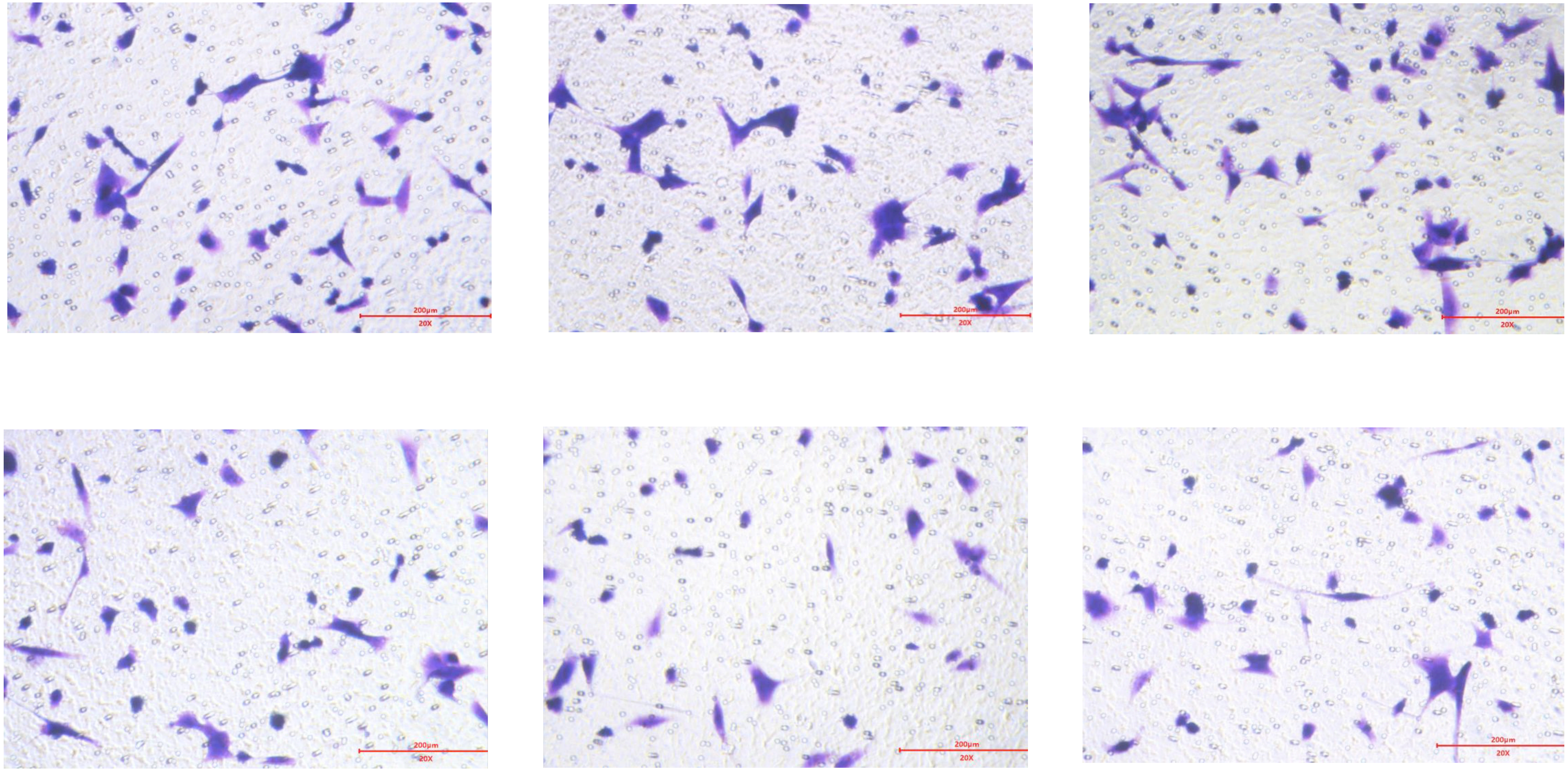

	**Cells/field**			SD	P value
U87-ARRB1-del exon13	80	70	74	5.0332	
U87-ARRB1-del exon13+2DG	50	40	58	9.0185	0.0132

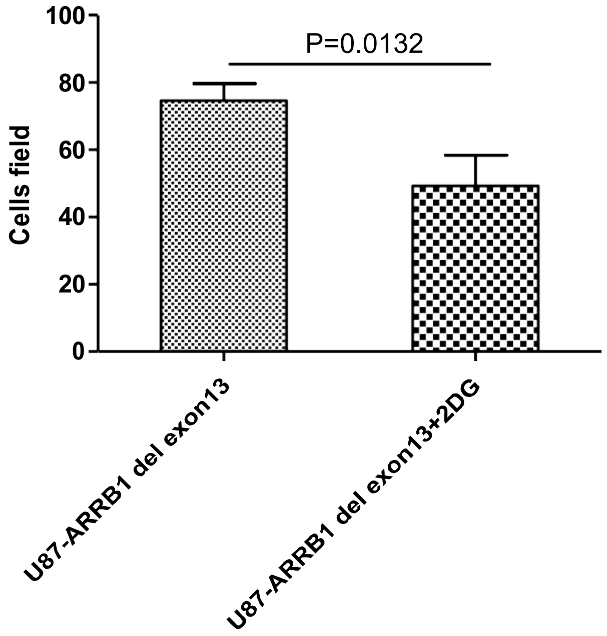

T98G-ARRB1-DEL-EXON13

T98G-ARRB1-DEL-EXON13+2-DG

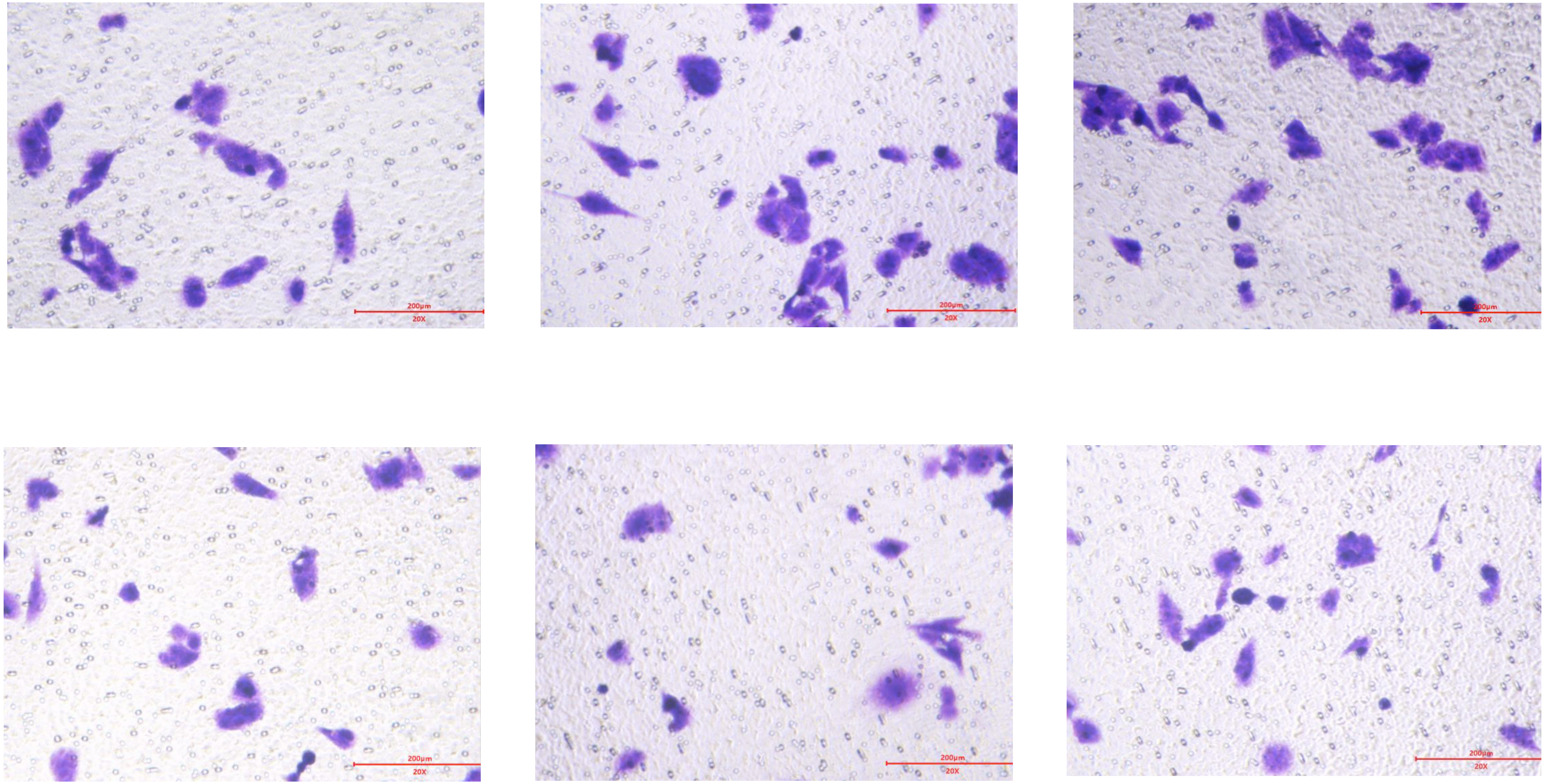

	**Cells/field**			SD	P value
T98G-ARRB1-del exon13	53	74	81	14.5717	
T98G-ARRB1-del exon13+2DG	37	30	42	6.0277	0.0223

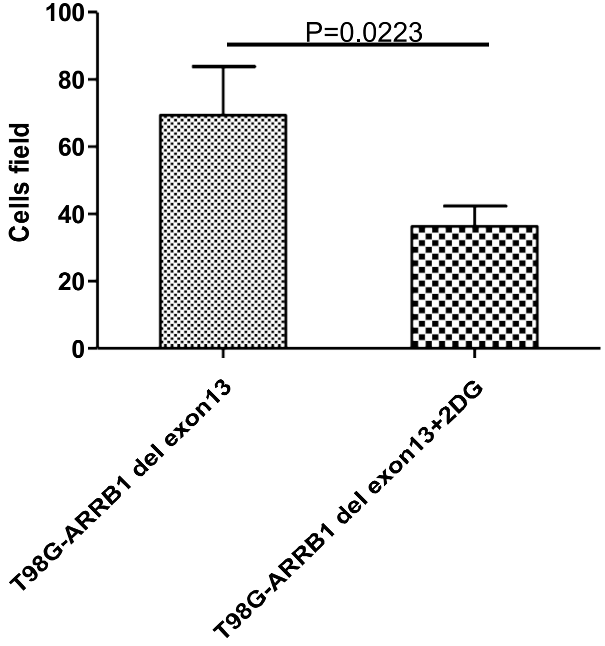

6

Colony formation

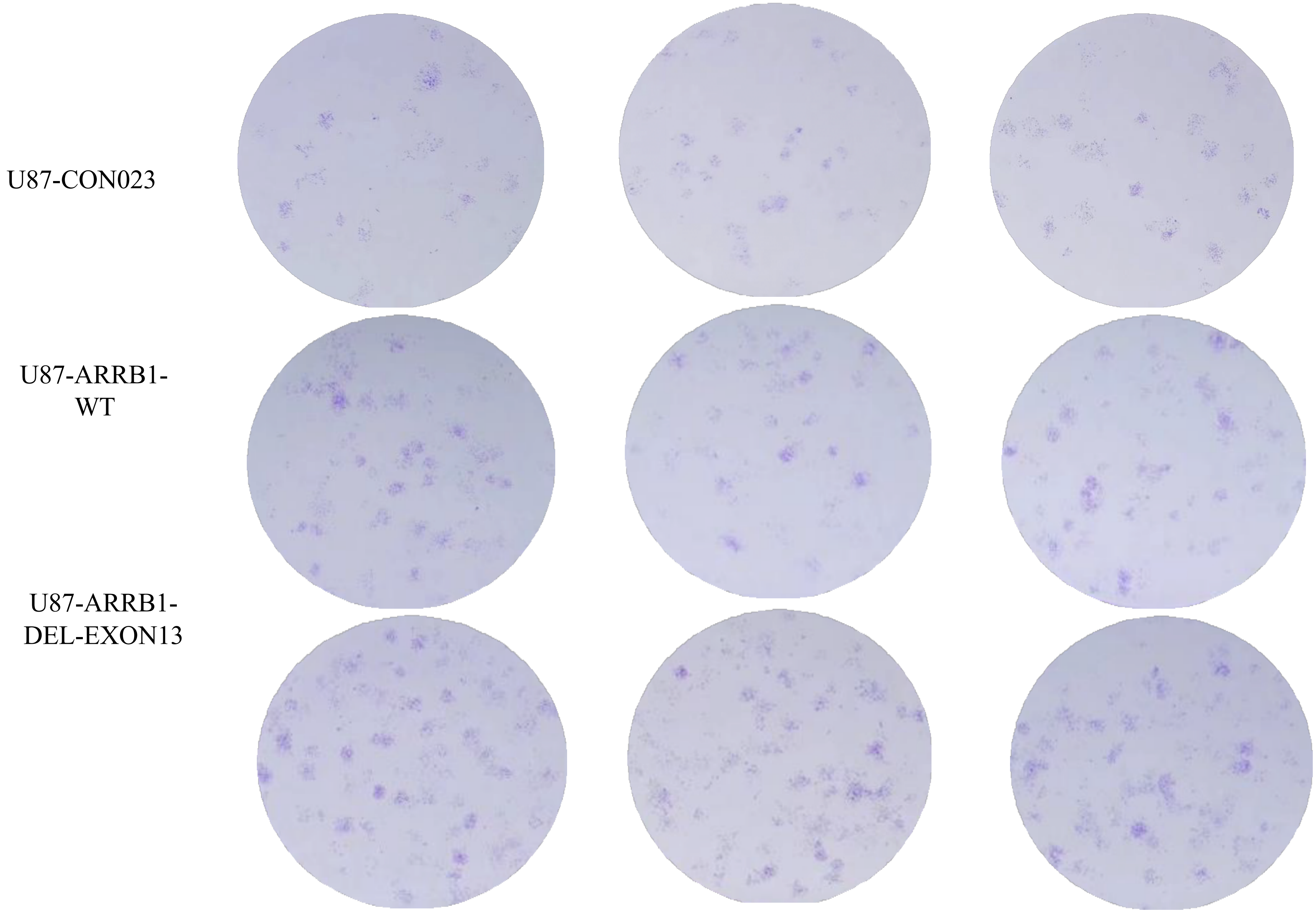
U87-CON023
U87-ARRB1-WT
U87-ARRB1-DEL-EXON13

U87-CON023

U87-ARRB1-WT

U87-ARRB1-DEL-EXON13

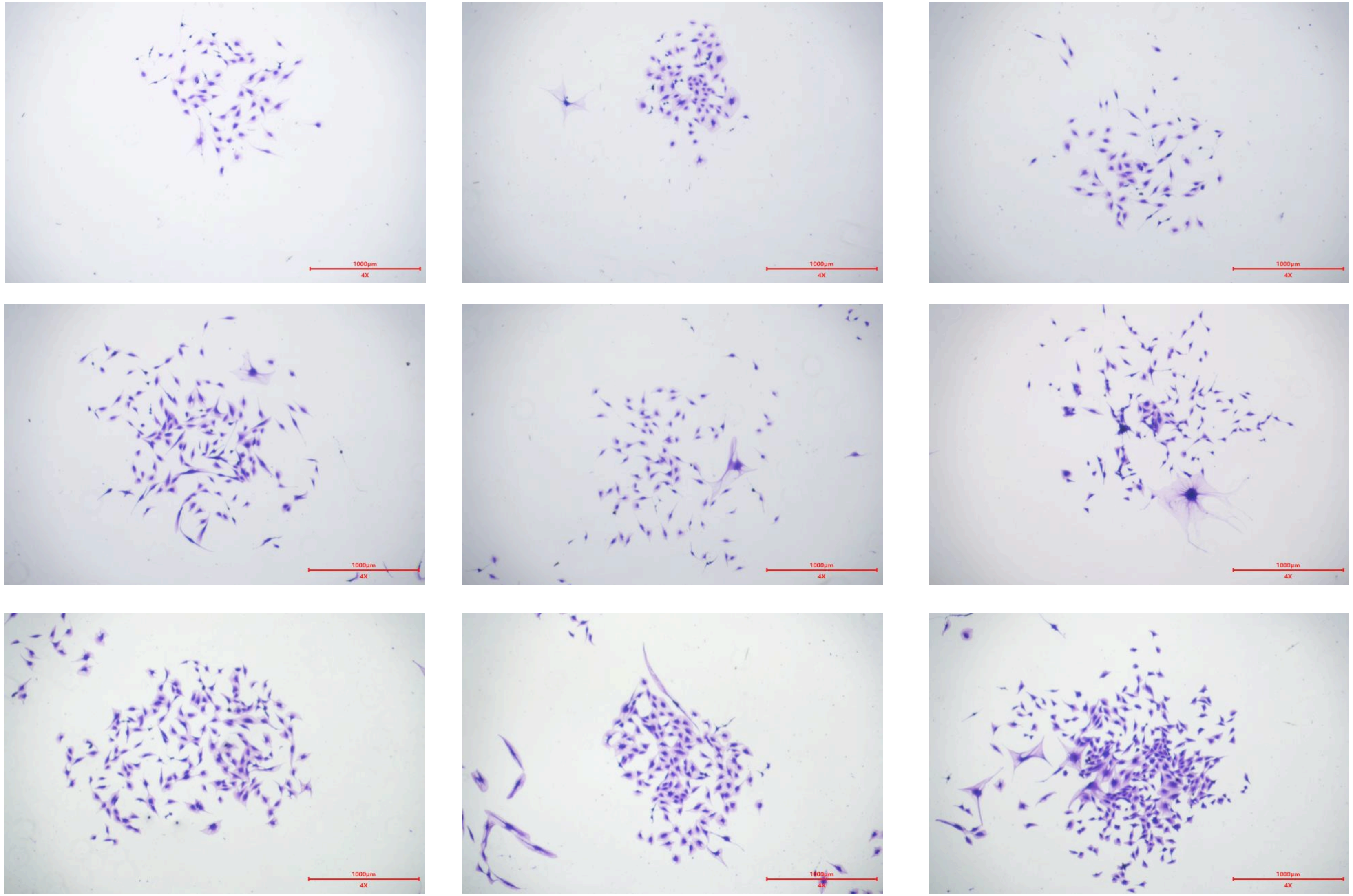

	Colony formation number			SD	P value
**U87-NC**	16	22	24	4.1633	
**U87-ARRB1-WT**	31	28	30	1.5275	0.0246
**U87-ARRB1-DEL-EXON13**	48	40	35	6.5574	0.0105

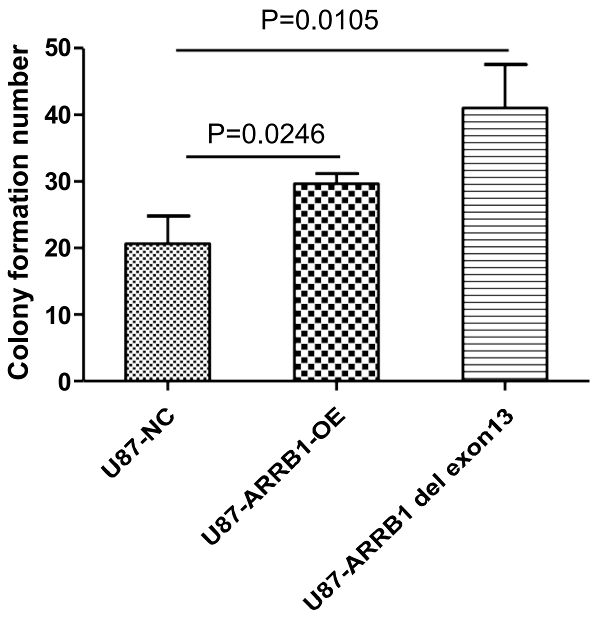

U87-ARRB1-DEL-EXON13

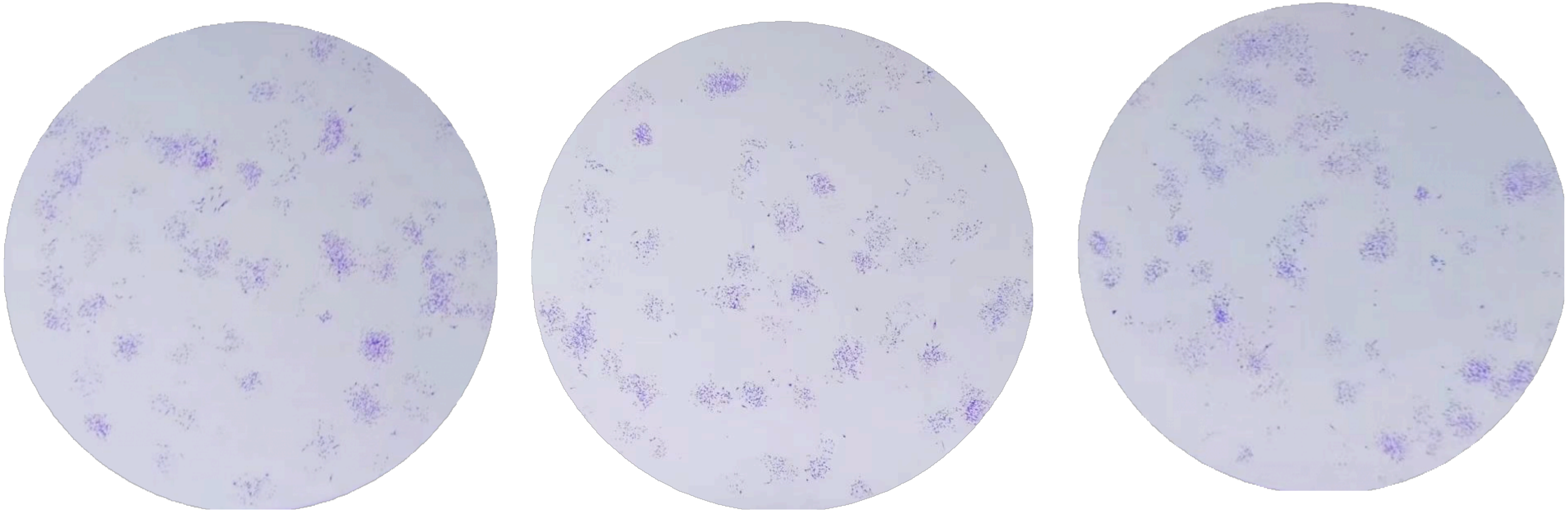

U87-ARRB1-DEL-EXON13+2-DG

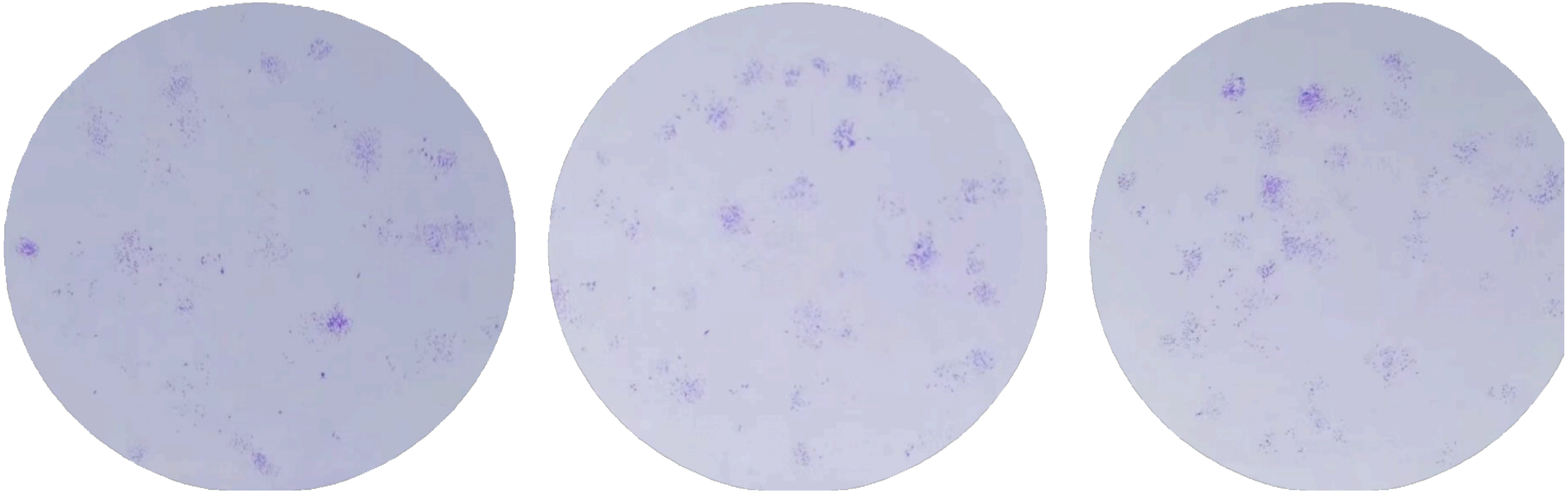

U87-ARRB1-DEL-EXON13

U87-ARRB1-DEL-EXON13+2-DG

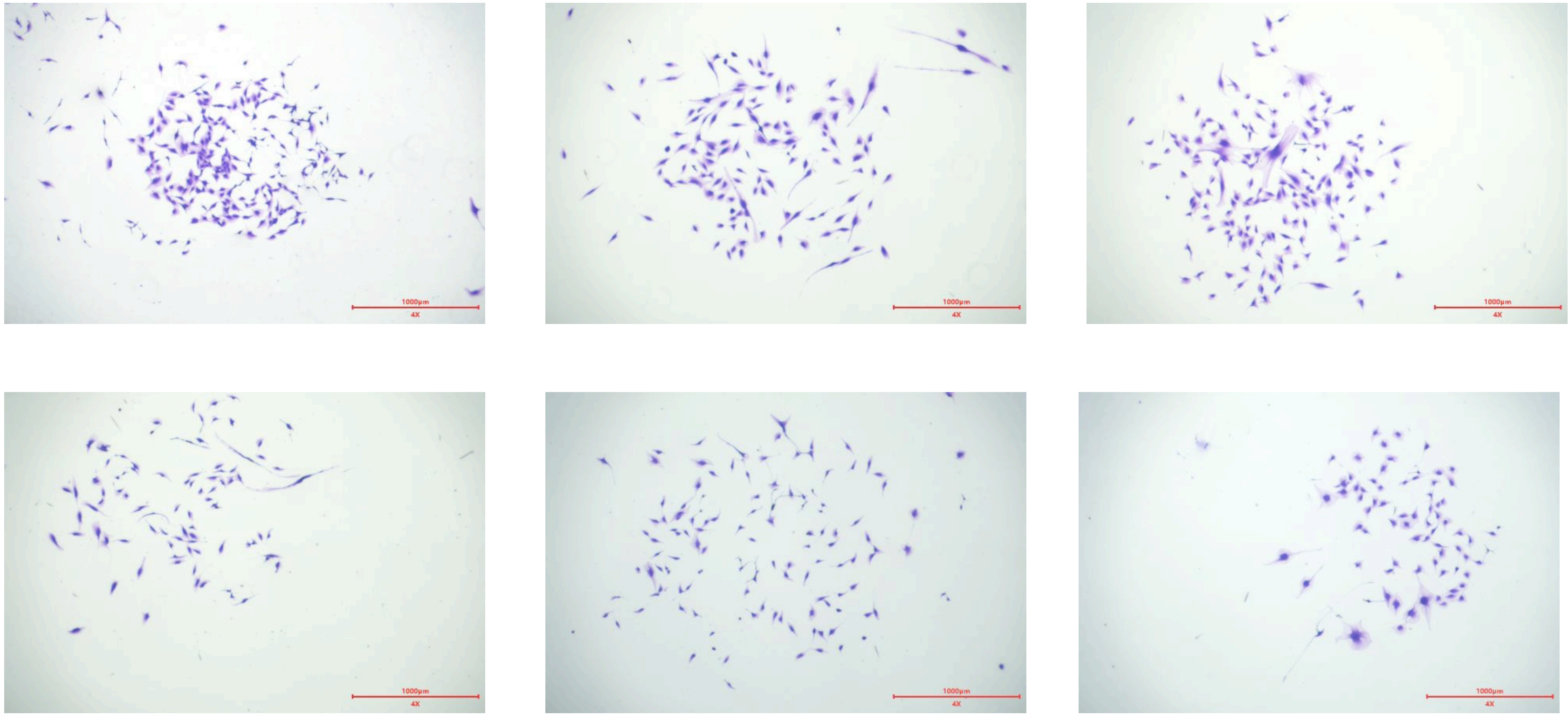

	Colony formation number			SD	P value
**U87-ARRB1-DEL-EXON13**	30	28	33	2.5166	
**U87-ARRB1-DEL-EXON13+2-DG**	12	18	14	3.0551	0.0024

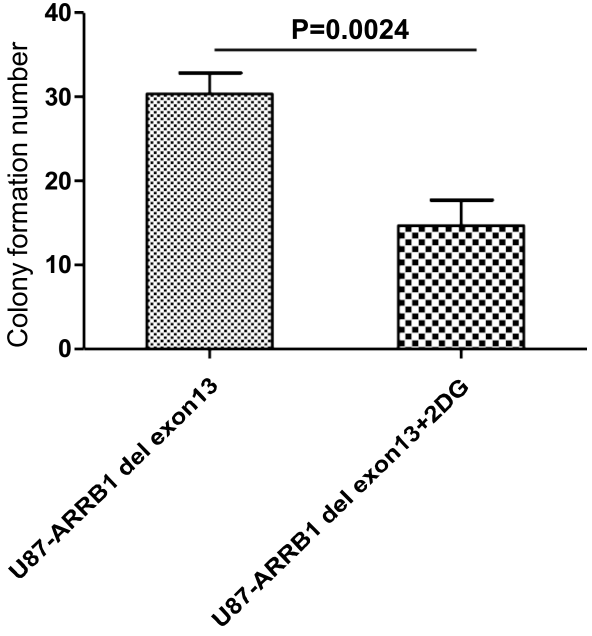

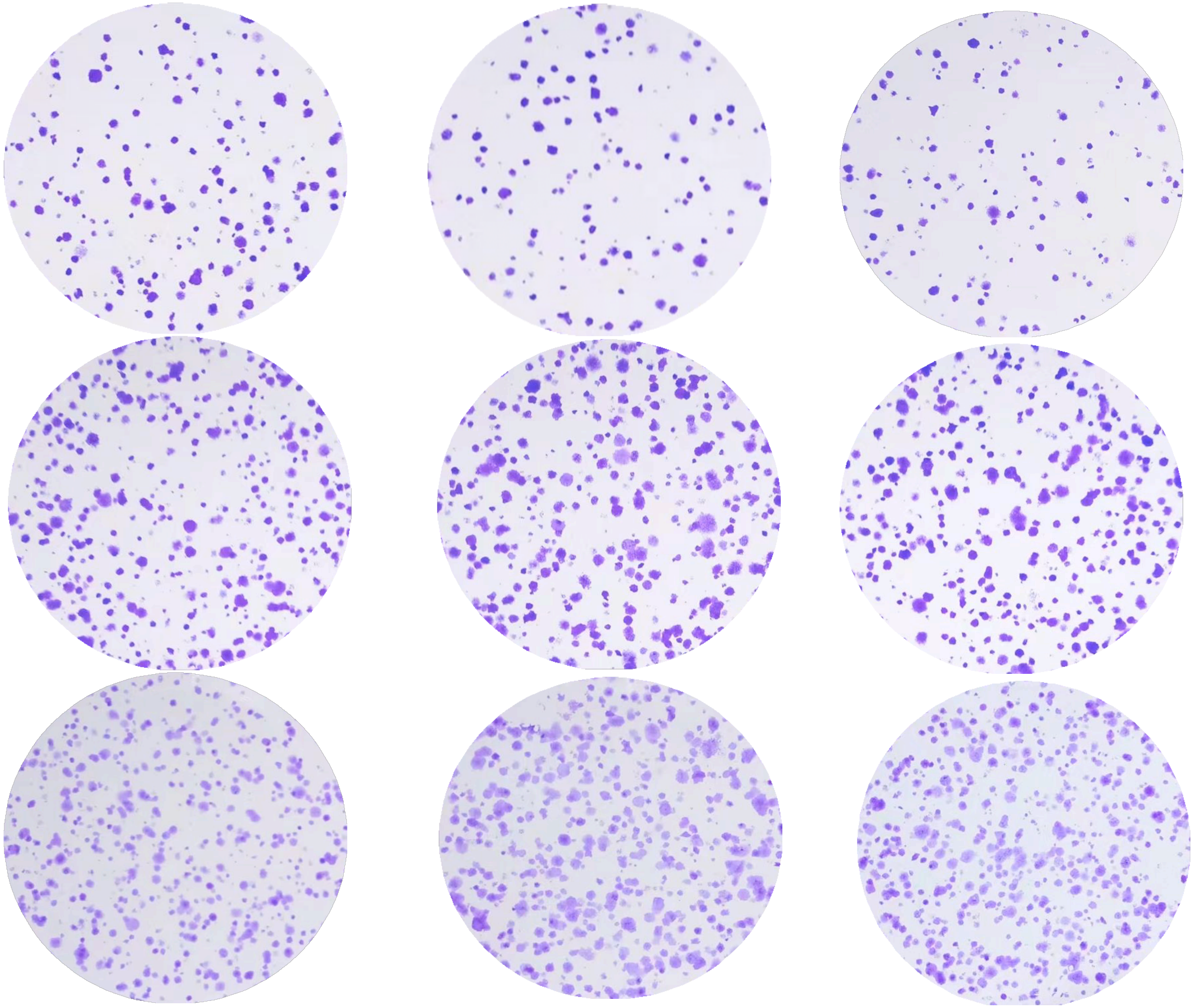

T98G-CON023

T98G-ARRB1-WT

T98G-ARRB1-DEL-EXON13

T98G-CON023

T98G-ARRB1-WT

T98G-ARRB1-DEL-EXON13

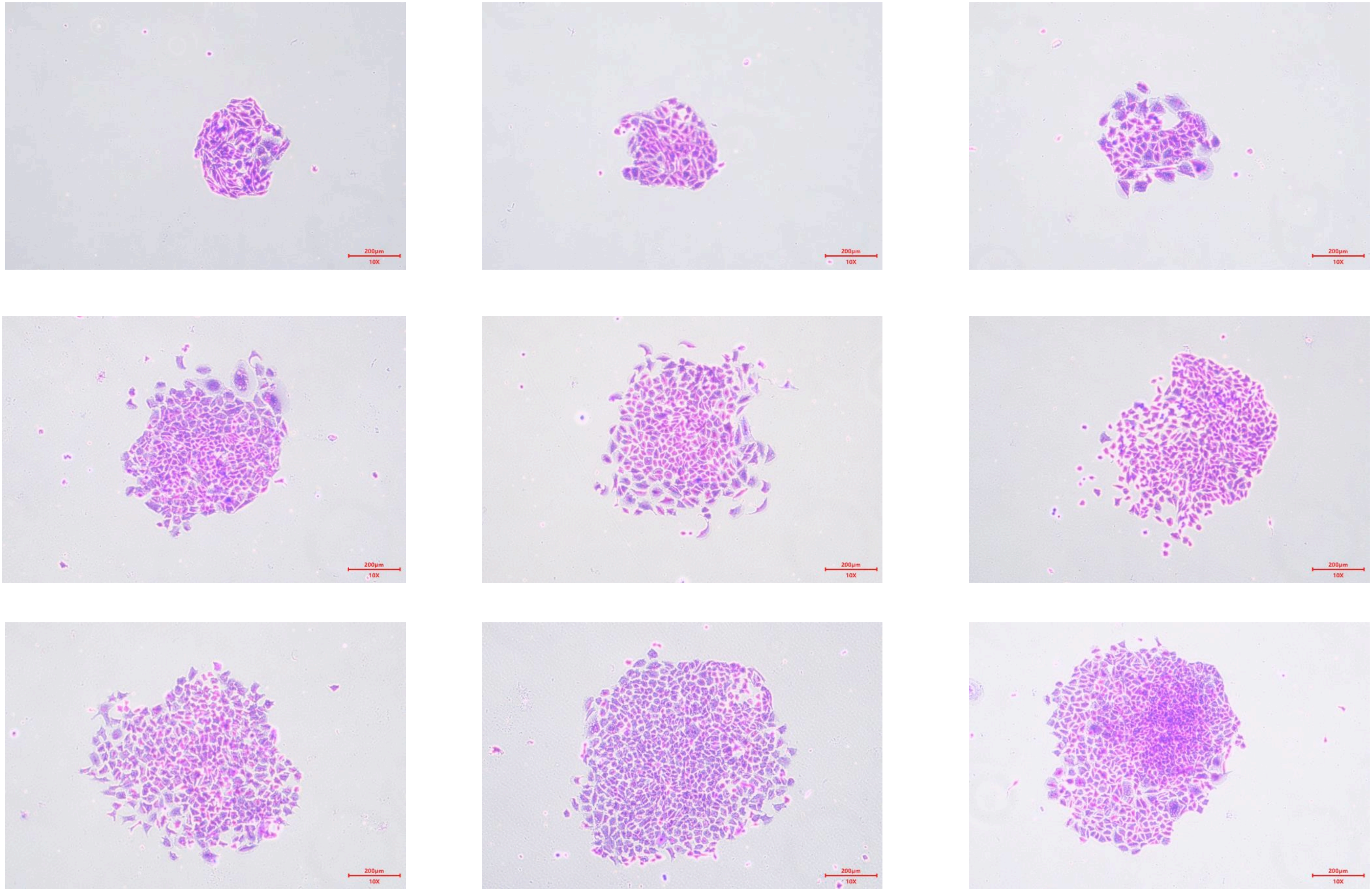

	Colony formation number			SD	P value
**T98G-CON023**	162	118	125	23.6432	
**T98G-ARRB1-WT**	338	359	330	14.9778	0.0002
**T98G-ARRB1-DEL-EXON13**	409	450	455	25.2389	0.0001

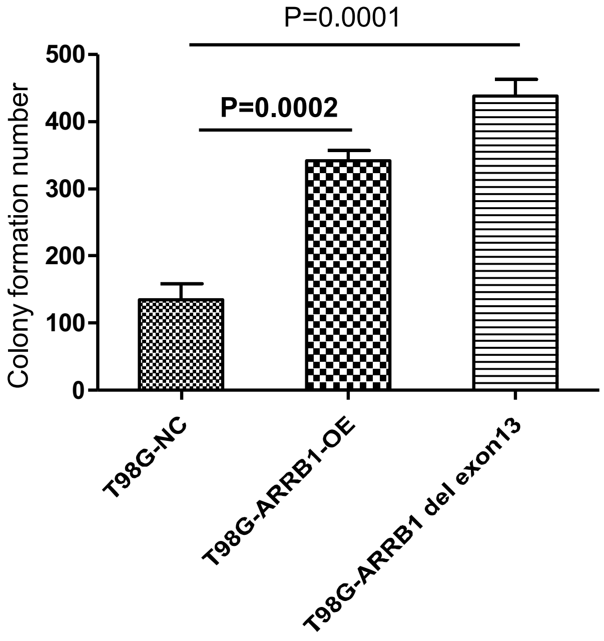

T98G-ARRB1-DEL-EXON13

T98G-ARRB1-DEL-EXON13+2-DG

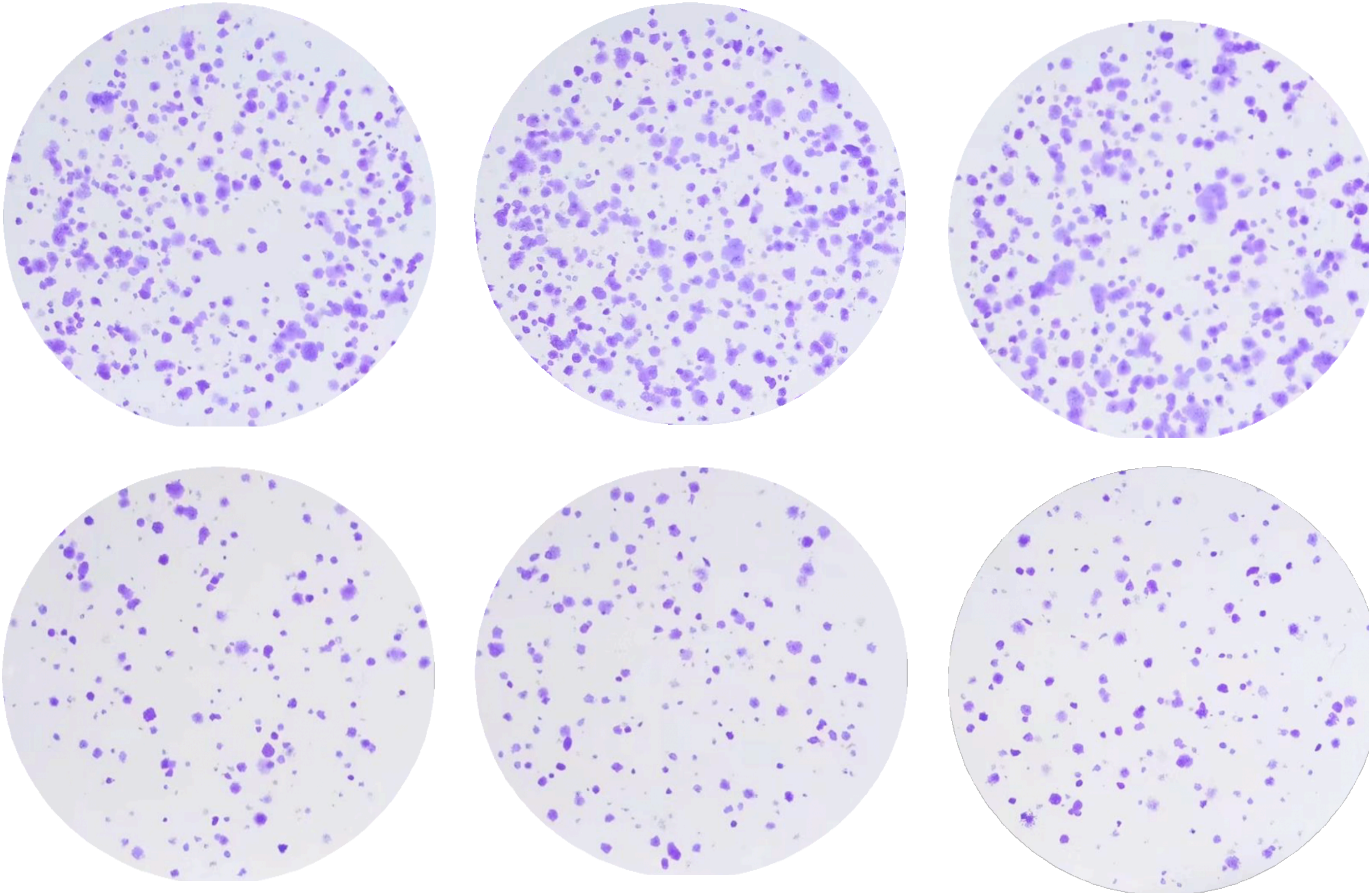

T98G-ARRB1-DEL-EXON13

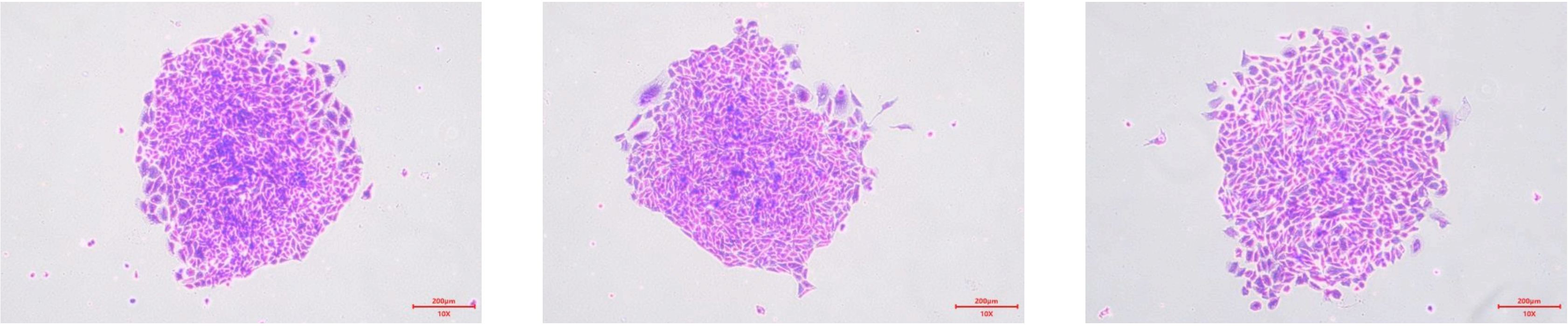

T98G-ARRB1-DEL-EXON13+2-DG

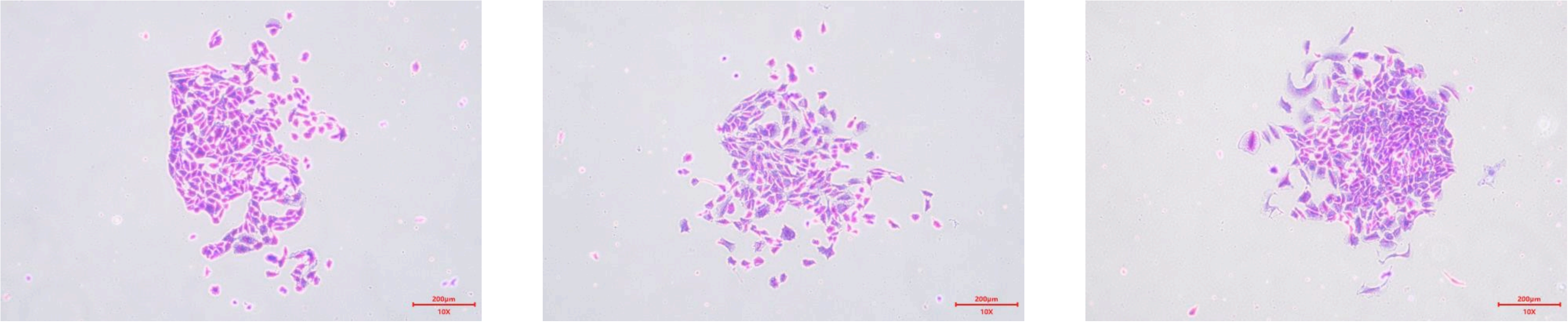

	Colony formation number			SD	P value
**T98G-ARRB1-DEL-EXON13**	440	470	419	25.6320	
**T98G-ARRB1-DEL-EXON13**+2-DG	151	143	136	7.5056	0.0000

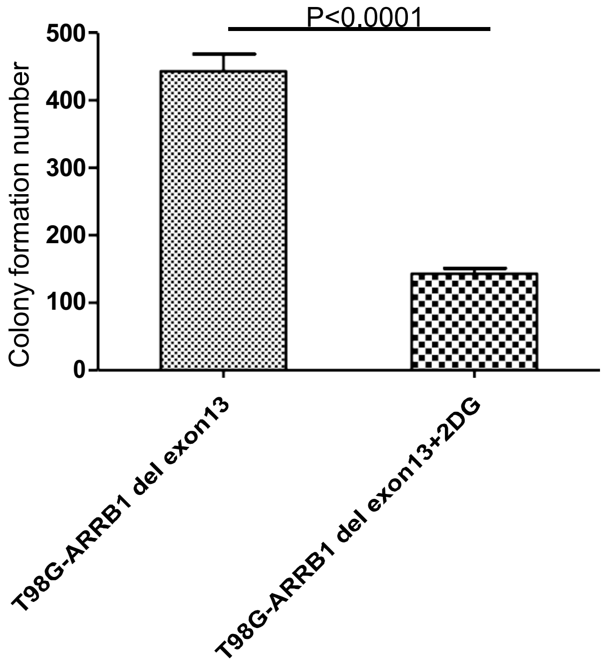

7 Pyruvic Acid Experiment

Cell	Value		
U87-NC	0.1051	0.1053	0.1063
U87-ARRRB1-WT	0.1223	0.1237	0.1193
U87-ARRB1-DEL-EXON13	0.1587	0.1551	0.1621
T98G-NC	0.1077	0.108	0.117
T98G-ARRRB1-WT	0.1532	0.1428	0.1426
T98G-ARRB1-DEL-EXON13	0.1919	0.1894	0.1894

	Pyruvic acid（μ g/104 cell)		
U87-NC	0.001617	0.001625	0.001668
U87-ARRRB1-WT	0.002356	0.002417	0.002227
U87-ARRB1-DEL-EXON13	0.003922	0.003767	0.004068
T98G-NC	0.001729	0.001741	0.002129
T98G-ARRRB1-WT	0.003685	0.003238	0.003229
T98G-ARRB1-DEL-EXON13	0.005349	0.005242	0.005242

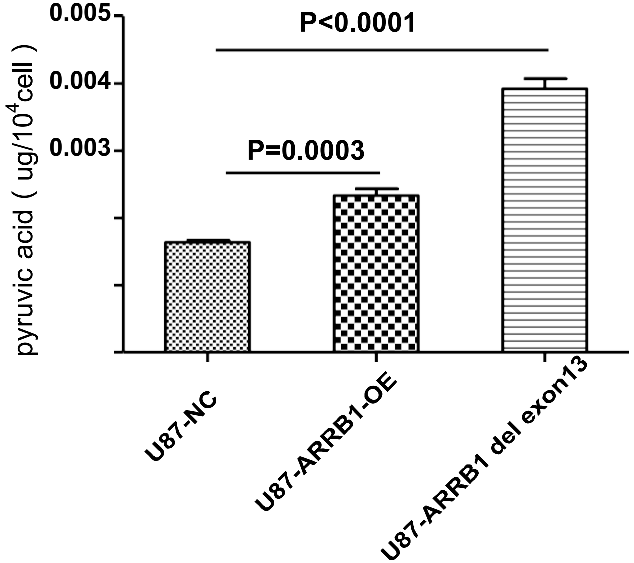
P<0.0001
P=0.0003
0.005
0.004
0.003
pyruvic acid (ug/10⁴cell)
U87-NC
U87-ARRB1-OE
U87-ARRB1 del exon13
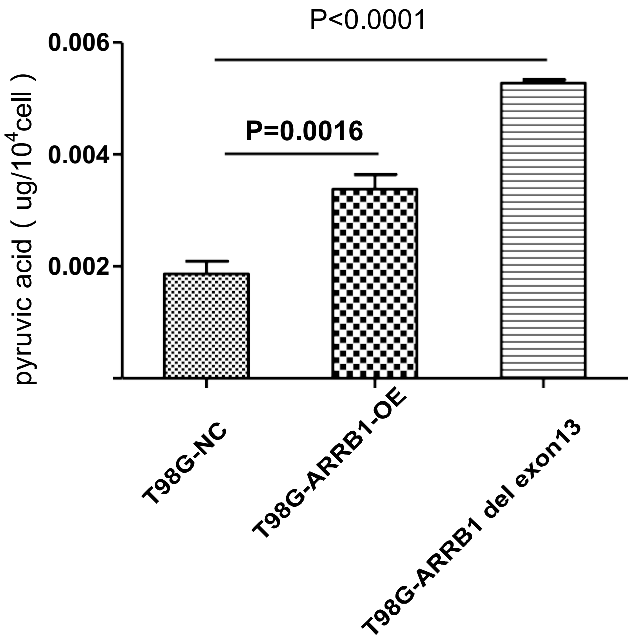
P<0.0001
P=0.0016
0.006
0.004
0.002
pyruvic acid (ug/10⁴cell)
T98G-NC
T98G-ARRB1-OE
T98G-ARRB1 del exon13
